# Balint groups for improving the ability of doctors and medical students to manage the doctor–patient relationship: a systematic review, quantitative meta-analysis and qualitative meta-synthesis of intervention studies

**DOI:** 10.1186/s12909-025-08072-z

**Published:** 2025-11-03

**Authors:** Luxinyi Xu, Xiaomei Cui, Yu Wang, Chuanchuan He, Lijun Dong, Dongmei Li, Yinglong Li, Yuan Yao, Liqin Shan, Zhengfen Xu

**Affiliations:** Jiaxing Maternity and Child Health Care Hospital, Jiaxing Women and Children’s Hospital, Wenzhou Medical University, Zhejiang, China

**Keywords:** Balint groups, Doctor–patient relationship, Systematic review, Meta-analysis, Meta-synthesis

## Abstract

**Objective:**

Balint groups are a crucial method for improving the relationship between medical students/doctors and patients. Nevertheless, no review has examined the effects of Balint groups in this regard. This study aimed to conduct quantitative meta-analyses and qualitative meta-syntheses based on a systematic review to provide references for improving doctor‒patient relationships.

**Methods:**

We searched six databases from inception through October 2024. Two reviewers independently conducted screening and quality assessment. Quantitative data were analyzed using meta-analysis methods with standard mean differences (SMDs) and 95% confidence intervals (CI) in RevMan 5.4.1, while qualitative results were summarized using meta-synthesis methods.

**Results:**

A total of 56 studies were included, including 45 quantitative studies and 11 qualitative studies. Two, fifty-one, and three studies were rated as having a low risk of bias, unclear risk or some concerns, and high risk of bias, respectively. Thirteen quantitative studies were included in the meta-analyses. Compared with those in the control group, participants in the Balint group had higher communication scores and empathy scores and lower anxiety scores (SMD = 1.26, 95% CI 0.97 to 1.56, *I*^2^ = 0%, five studies; SMD = 2.40, 95% CI 1.31 to 3.49, *I*^2^ = 96%, six studies; SMD = -0.79, 95% CI -1.39 to -0.19, *I*^2^ = 71%, three studies). Participants who received the Balint intervention had significantly lower burnout scores in emotional exhaustion and reduced personal accomplishment among healthcare workers post-intervention compared with pre-intervention (SMD = -1.62, 95% CI -3.21 to -0.03, *I*^2^ = 88%, three studies; SMD = -1.22, 95% CI -2.26 to -0.17, *I*^2^ = 74%, three studies), while no significant change was saw in cynicism (SMD = -0.90, 95%, CI -1.91 to 0.10, *I*^2^ = 75%, three studies). The meta-synthesis results of 11 qualitative studies show that Balint groups have a positive effect on doctors’ doctor–patient communication, empathy, psychological adjustment, and team cooperation abilities.

**Conclusion:**

Balint groups may contribute to improving doctor‒patient relationships. We suggest caution and advocate for multicenter large-sample randomized controlled trials with low risk-of-bias design to avoid evidence bias.

**Supplementary Information:**

The online version contains supplementary material available at 10.1186/s12909-025-08072-z.

## Introduction

Conflict between physicians and patients is a serious problem that affects society, the government, patients, and doctors. China still lacks medical resources to meet the growing health demands [1]. It may be difficult for physicians and patients to establish a harmonious relationship; sometimes, even quarrels and violent incidents occur. The root cause lies in the physician–patient mistrust and knowledge asymmetry [2].

How to restore doctor–patient trust is a significant issue in the present healthcare reform. Researchers are increasingly focusing on the doctor‒patient relationship, with Balint groups being one of the most prominent methods used today. The Balint group was developed by psychiatrist Michael Balint and his wife Enid Balint in the 1950s. Over twenty countries, including China, have established Balint associations to address doctor‒patient conflicts [3, 4]. Through case discussions, this method can help physicians view cases from various angles, better empathize with patients, enhance communication and self-reflection abilities, and alleviate the negative emotions in their medical work [5–7]. 

No researchers have yet to systematically evaluate the role of Balint groups in improving doctor–patient relationships. Therefore, on the basis of a systematic review of the relevant studies, we conducted meta-analyses and meta-syntheses of quantitative and qualitative results, respectively. This paper summarizes the studies on the effects of Balint groups on improving doctor–patient relationships among doctors or medical students. Our findings could provide references for improving doctors’ ability to communicate with their patients, the mental health of doctors, and doctor–patient relationships.

## Methods

We reported our review by following the Preferred Reporting Items for Systematic Reviews and Meta-Analyses (PRISMA) statement [8]. This study is not registered in PROSPERO.

### Search strategy

Our searches were conducted on 25/10/2024 in PubMed, EMBASE, the Cochrane Library, the China National Knowledge Infrastructure (CNKI), WanFang, and the China Science and Technology Journal Database (CSTJ) (from inception through October 2024). We used Boolean operators (‘AND’ and ‘OR’) to combine both MeSH terms and free text (including Balint, Balint group, Balint therapy, doctor‒patient interaction, physician‒patient interaction, doctor‒patient communication, and physician‒patient communication, etc.) (see Supplementary Appendix 1 for detailed search strategies). Only full-text articles in English or Chinese were included. Duplicate records were excluded.

### Study selection and eligibility criteria

Two reviewers (L.X. and X.C.) independently screened the title, abstract and full text of studies. In case of disagreement, the final decision was made in consultation with a third reviewer (Z.X.). Studies were screened using Noteexpress.

To be included, studies had to meet the following criteria under the Population, Intervention, Comparison, Outcome, and Study type (PICOS) principle:

- Population: The target population was clinicians, resident doctors, or clinical medical students. If the intervention population includes individuals other than the aforementioned groups, this article should be included. The article should be excluded if the intervention study only includes nurses, patients, or other individuals.
- Intervention: This study focused on Balint groups. Therefore, studies on any intervention that uses Balint groups were included, regardless of whether other effective intervention measures were combined or not. Studies that did not use the Balint group for intervention were excluded.
- Comparison: Studies including a blank control group, placebo, or other effective intervention. Single-arm studies with pre-post designs without control groups were also included.
- Outcomes: For quantitative studies, any indicators related to doctor‒patient interactions, such as doctor‒patient communication ability, empathy, anxiety, depression, and coping style, were included; for qualitative research, structured or unstructured interviews and qualitative analysis were included. Studies without reported results directly or indirectly related to the doctor‒patient relationship and those without reporting key outcome data were excluded.
- Study type: Interventional studies, including randomized controlled trials (RCTs), cluster randomized trials, crossover trials, and single-arm trials with pre-post designs were included. Non-experimental research designs (i.e., cross sectional studies, cohort studies), reviews, abstracts, and conference articles were excluded.

### Data abstraction

Two reviewers (L.X. and X.C.) extracted the data independently, and any disagreements were resolved by discussing the data and consulting with a third reviewer (Z.X.). We collected the following information from each study: (a) first author; (b) year of publication; (c) research object; (d) sample size; (e) sampling and grouping methods; (f) intervention design (method, frequency, and duration); (g) outcome evaluation method (index, time point); (h) outcome data: for quantitative studies, averages and standard deviations were collected; for qualitative research, verbatims described by the interviewees and texts in the qualitative analysis regarding the effect of Balint groups were extracted.

### Quality assessment

The same two reviewers (L.X. and X.C.) independently rated the risk of bias of each study for every domain, The “overall” risk was rated as “high risk”, “some concerns”, or “low risk”, concerning the randomization process, allocation concealment, blinding, measurement, attrition, selective reporting, and other types of bias. Version 2 of the Cochrane Risk of Bias Tool (RoB 2) [9] was used to assess randomized controlled trials, cluster randomized trials, crossover trials. The adapted Newcastle‒Ottawa Scale (NOS) [10] was used for single-arm trials. To make the quality assessment of single-arm trials with pre-post designs more suitable, we removed the questions about the control group and one of the entries used to assess the outcome of the NOS. All discrepancies were discussed with a third reviewer (Z.X.) until a consensus was reached.

### Statistical analysis

Based on the nature of the outcome measures (quantitative or qualitative), we classified the included studies into quantitative and qualitative studies.

For quantitative studies, i.e., studies using quantitative data, we performed a meta-analysis when data from at least three studies with the same outcome variables were available. Data from the reports with the longest interventions were used for meta-analyses. We calculated standardized mean differences (SMDs) complemented by 95% confidence intervals (CI) for continuous outcomes, with α = 0.05 as the significance level. SMD is calculated by dividing the pooled mean difference by the standard deviation, which can be used to compare studies that measure outcomes using different scales [11]. The values of SMD and its upper and lower limits of 95% CI all > 0 or < 0 indicate that the effect value of the experimental group/post-intervention is statistically different from that of the control group/pre-intervention. Heterogeneity was quantified by the χ^2^ test. When *I*^2^ was < 50%, the heterogeneity was considered acceptable, and the fixed effects model was used for the meta-analysis. When *I*^2^ was ≥ 50%, the heterogeneity was considered significant, and the random effects model was used for analysis. When *I*^2^ was > 80%, subgroup analyses were performed to explore sources of heterogeneity. The grouping criteria (including participants, language, region, intervention measures, and risk of bias, if possible) were determined based on the included studies. Sensitivity analyses were conducted by omitting each study separately to examine the impact of each study on the overall results. Publication bias was explored by observing the funnel plot. The quality of the evidence of this study was evaluated by the Grades of Recommendation, Assessment, Development, and Evaluation (GRADE) using GRADEpro (https://www.gradepro.org/). Statistical analyses were performed in RevMan 5.4.1.

For qualitative studies, i.e., studies using interviews or qualitative analysis, we performed qualitative meta-syntheses. The aggregative synthesis method of the Joanna Briggs Institute (JBI) Evidence-based health care center was applied to summarize the included contents (results) one by one into sub-themes (categories) and themes (integration results). Based on carefully reading the full text, we extracted the original texts related to the direct or indirect role of the Balint group in improving the doctor‒patient relationship and formed results one by one, then summarized similar results as sub-themes and finally integrated them into themes.

## Results

### Study selection

**Fig. 1:**
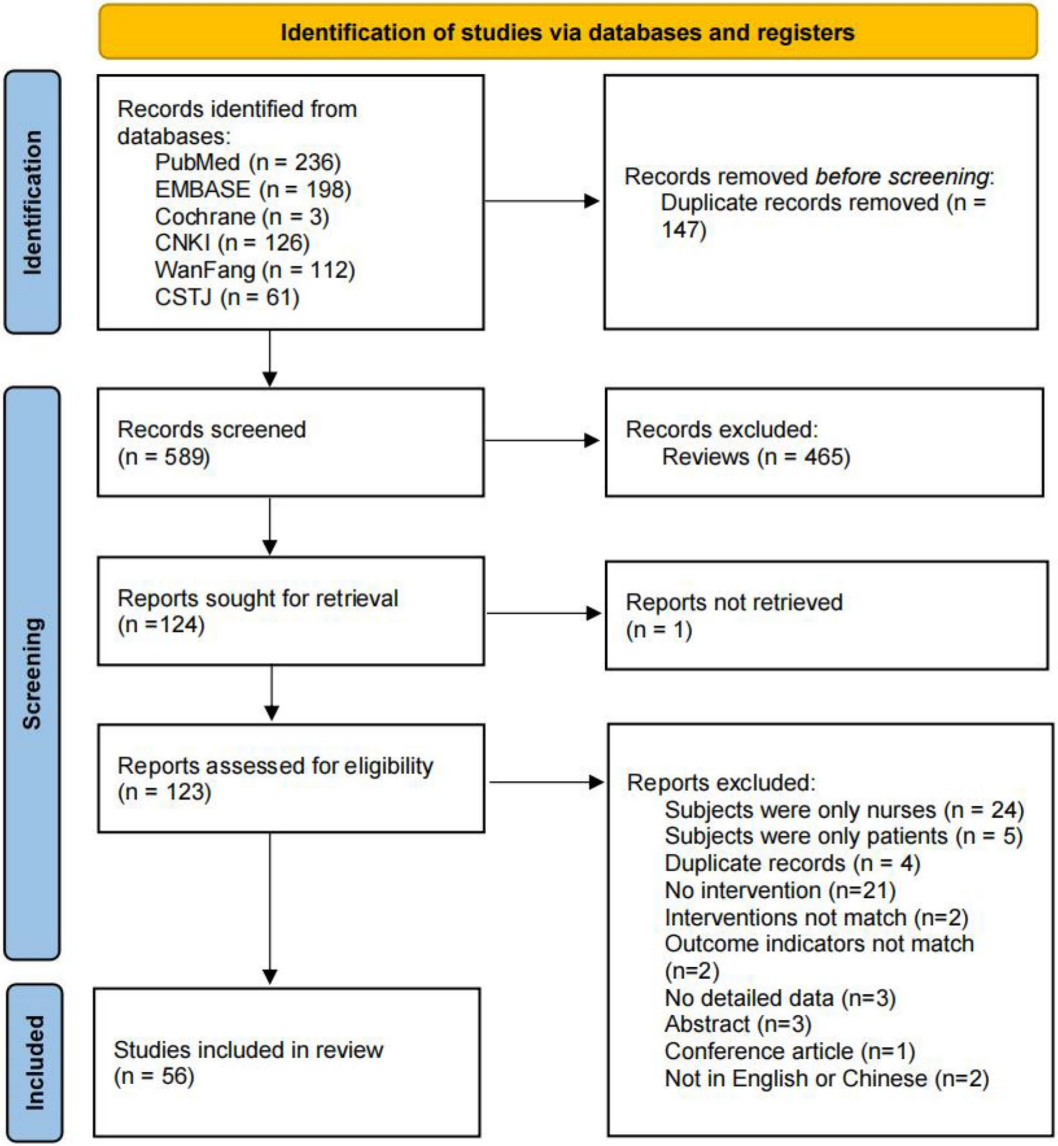
PRISMA flow chart of study identification and screening

Among the 736 records identified from the databases, 56 studies were included (Fig. 1). Of these studies, 37 were published in Chinese, and 19 were published in English.

### Study characteristics

Tables 1 and 2 show the characteristics of the studies included in the systematic review, whereas Table 3 lists the outcome measurement methods used in each study.

**Table 1:** Characteristics of studies included in the systematic review (controlled studies)

Study ID	Country	Study design	Sample size	Population	Study arms		Implementation frequency, times of Balint	Study duration (months)	Risk of bias
					Experimental group	Control group			
[12]	China	RCT	24	Clinical medical students	Balint group	None	Once every two weeks, 10	5	some concerns
[13]	China	RCT	60	Clinical medical students	Balint group	None	Weekly, 20	5	some concerns
[14]	China	RCT	20	Resident doctors	Balint group	None	Once every two weeks, 8	4	some concerns
[15]	China	RCT	120	Clinical medical students	Balint group	None	Weekly, 4	1	some concerns
[16]	China	RCT	60	Resident doctors	Balint group	None	Once or twice every week, 4	Not reported	some concerns
[17]	China	RCT	74	Resident doctors	Miller pyramid theory + Balint group	None	Once every two weeks, not reported	Not reported	some concerns
[18]	China	RCT	39	Clinical medical students	Balint group	Consolation control group: propaganda and education; Blank control group: none	Weekly, 8	2	some concerns
[7]	France	RCT	362	Clinical medical students	Group 1: Balint group	Group 2: narrative medicine; Group 3: none	Weekly, 7	3	low risk
[19]	China	RCT	120	Physicians, nurses	Balint group + Music therapy	Music therapy	Weekly, 4	1	some concerns
[20]	China	RCT	16	Resident doctors	Balint group	Exercise prescription intervention	Once every two weeks, 6	3	some concerns
[21]	China	RCT	60	Physicians, nurses	Balint group	None	Once every two months, 12	24	some concerns
[22]	China	RCT	36	Physicians, nurses	Balint group	Consolation control group: network psychological self-help intervention; Blank control group: none	Weekly, 6	1.5	some concerns
[23]	USA	RCT	14	Resident doctors	Balint group + Behavioral health curriculum	Behavioral health curriculum	Once every two weeks, 18	9	some concerns
[24]	USA	RCT	16	Resident doctors	Balint group	None	Once every two weeks, 12	6	some concerns
[25]	China	RCT	90	Clinical medical students	Group 1: Balint group	Group 2: publicity and education; Group 3: doctor–patient relationship learning	Once every two weeks, 12	6	some concerns
[26]	China	RCT	60	Clinical medical students	Balint group	None	Monthly, 6	6	some concerns
[27]	UK	RCT	30	Clinical medical students	Group 1: Balint group	Group 2: psychotherapy program; Group 3: none	Weekly, 12	3	some concerns
[28]	China	RCT	20	Physicians	Balint group	None	Not reported	Not reported	some concerns
[29]	Israel	RCT	34	Physicians	a breaking bad news (BBN) training program	Balint group	Not reported	Not reported	high risk
[30]	China	RCT	36	Resident doctors	Balint group	None	Not reported, 10	6	some concerns
[31]	France	RCT	163	Clinical medical students	Balint group	None	Weekly, 10	5	some concerns
[32]	France	RCT	299	Clinical medical students	Balint group	None	Weekly, 7	over 2 months	low risk
[33]	China	RCT	30	Clinical medical students	Balint group	None	Weekly, 10	2.5	some concerns
[34]	China	RCT	40	Resident doctors	Balint group	None	Once every week or every two weeks, not reported	4.5	some concerns
[35]	China	RCT	60	Resident doctors	Balint group + Doctor–patient communication course	Doctor–patient communication course	Not reported,2	Not reported	some concerns
[36]	China	Cluster	56	Resident doctors	Balint group	None	Twice every week, 8	4	high risk
[37]	China	Cluster	58	Clinical medical students	Balint group + Doctor–patient relationship learning	Doctor–patient relationship learning	Not reported, 4	Not reported	some concerns
[38]	USA	Crossover	28	Clinical medical students	Balint group (in the first 6 months)	Balint group (in the next 6 months)	Once every two weeks, 12	6	some concerns

**Table 2:** Characteristics of studies included in the systematic review (single-arm studies)

Study ID	Country	Study design	Sample size	Population	Intervention	Implementation frequency, times of Balint	Study duration (months)	Risk of bias
[39]	China	Single-arm	155	Clinical medical students	Balint group	Weekly, 20	5	unclear
[40]	China	Single-arm	75	Resident doctors	Balint group	Once every two weeks, 6	3	unclear
[41]	China	Single-arm	30	Physicians	Balint group	Weekly, not reported	6	unclear
[42]	China	Single-arm	20	Physicians, nurses	Balint group	Once every two weeks, 8	4	unclear
[43]	China	Single-arm	76	Resident doctors	Balint group	Not reported	Not reported	unclear
[44]	China	Single-arm	35	Physicians, nurses	Balint group	Once every two weeks, 6	3	unclear
[45]	China	Single-arm	54	Physicians, nurses	Balint group	Weekly, 6	1.5	high
[46]	China	Single-arm	30	Clinical medical students	Balint group	Once or twice every two months, not reported	Not reported	unclear
[47]	China	Single-arm	104	Clinical medical students	Balint group	Not reported	Not reported	unclear
[48]	UK	Single-arm	16	Clinical medical students	Balint group	Weekly, 5	1.25	unclear
[49]	China	Single-arm	68	Clinical medical students	Balint group	Not reported, 3	Not reported	unclear
[50]	Australia	Single-arm	20	Clinical medical students	Balint group	Weekly, 6	1.5	unclear
[51]	Australia	Single-arm	42	Clinical medical students	Balint group	Not reported	Not reported	unclear
[52]	Netherlands	Single-arm	22	Physicians	Balint group	Not reported	Not reported	unclear
[53]	China	Single-arm	18	Resident doctors	Balint group	Monthly, 26 (12 times per person)	12	unclear
[54]	China	Single-arm	20	Resident doctors	Balint group + Figure sculpture	Not reported, 16	Not reported	unclear
[55]	China	Single-arm	54	Resident doctors	Balint group	Once every two weeks, 30 (5–6 times per person)	3	unclear
[56]	China	Single-arm	136	Physicians, nurses	Balint group	Monthly, 17	Not reported	unclear
[57]	China	Single-arm	160	Physicians, nurses	Balint group	Not reported	Not reported	unclear
[58]	Singapore	Single-arm	26	Physicians	Balint group (online)	Not reported	Not reported	unclear
[59]	China	Single-arm	13	Clinical medical students	Balint group	Weekly, 6	3	unclear
[60]	Finland	Single-arm	9	Clinical medical students	Balint group	Weekly, ‘five people group’ 10 times per person, ‘four people group’ 5 times per person	Not reported	unclear
[61]	New Zeland	Single-arm	6	Clinical medical students	Balint group	Weekly, 6	1.5	unclear
[62]	China	Single-arm	30	Physicians, nurses, medical technicians	Balint group	Monthly, 6 times per person	11	unclear
[63]	USA	Single-arm	18	Resident doctors	Balint group	Weekly, 24	6	unclear
[64]	China	Single-arm	30	Physicians	Balint group	Not reported, 10	12	unclear
[65]	China	Single-arm	55	Physicians, clinical medical students	Balint group	Not reported, 10	Not reported	unclear
[66]	USA	Single-arm	55	Resident doctors	Balint group	Not reported	12	high

**Table 3:** Outcome measurement of the included studies

No	Outcome measurement	Study ID	Number of studies
1	Other self-designed questionnaires	[16, 17, 25–29, 31, 34, 35, 43, 48–58]	22
2	SEGUE (Set Elicit Give Understand End framework)	[12–17, 35, 36, 39, 40, 43]	11
3	Jefferson’s Empathy Scale	[7, 12–14, 18, 32, 35, 37, 43, 66]	10
4	Interview	[24, 56–63]	9
5	Maslach Burnout Inventory General Survey (MBI-GS), Maslach Burnout Inventory-Human Services Survey (MBI-HSS)	[21, 30, 41–43, 66]	6
6	Self-rating Anxiety Scale (SAS)	[19, 21, 22, 42, 44]	5
7	Self-rating Depression Scale (SDS)	[20–22, 42, 44]	5
8	Coping Strategy Questionnaire (CSQ)	[21, 22, 37, 44, 45]	5
9	Psychological Medicine Inventory (PMI)	[23, 24, 66]	3
10	Liverpool Communication Skills Assessment Scale (LCSAS)	[15, 46]	2
11	Interpersonal Trust Scale (ITS)	[33, 46]	2
12	Qualitative analysis of the discussion content	[64, 65]	2
13	Doctor–patient Communication Quality Evaluation Scale	[15]	1
14	Pittsburgh Sleep Quality Index (PSQI)	[22]	1
15	Emotional Intelligence Scale (EIS)	[37]	1
16	Physician’s Belief Scale	[38]	1
17	General Well-Being Schedule Scale (GWB)	[44]	1
18	Symptom Checklist 90 (SCL-90)	[44]	1
19	Clinical Communication skills Scale	[45]	1
20	Sense of Security Questionnaire (SQ)	[46]	1
21	Communication Skills Attitude Scale (CSAS)	[47]	1
22	Minnesota Satisfaction Questionnaire (MSQ)	[30]	1
23	Interpersonal reactivity index (IRI)	[31]	1
24	Consultation And Relational Empathy Measure (CARE)	[32]	1
25	College Students’ Empathy Ability questionnaire	[33]	1

A total of 56 studies involving 3364 healthcare workers were included. Of these included studies, 28 were single-arm trials, 25 were RCTs, 2 were cluster randomized trials, and 1 was a crossover trial. China had the most studies (*n =* 39), followed by the United States (*n =* 5) and France (*n =* 3). Twenty-three studies included clinical medical students, 16 included physicians or medical personnel (including physicians), and 17 included resident doctors. The majority of the included studies used self-designed questionnaires (*n =* 22) as the assessment tool, followed by interviews or qualitative analysis of the discussion content (*n =* 11) and the SEGUE scale (*n =* 11).

### Risk of bias

The risk of bias in the included studies was low for two studies, high for three studies, and unclear or concerning for the remainder (Table 1 and Table 2). Details can be found in Supplementary Appendix 2.

### Meta-analysis

In this study, a total of 13 quantitative studies were included in the meta-analyses.

#### SEGUE score (communication score)

**Fig. 2:**
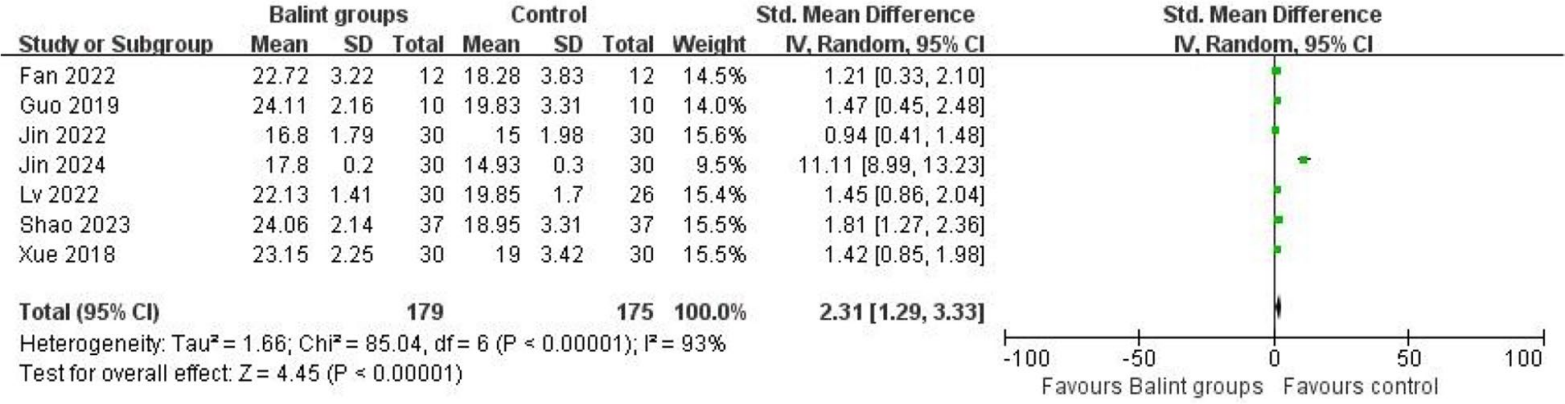
Effect of Balint groups including co-interventions SEGUE score. The comparative result of *IV* Inverse variance, random effects meta-analysis between the Balint group and control group. *SD* Standard deviation, *CI* Confidence interval

**Fig. 3:**
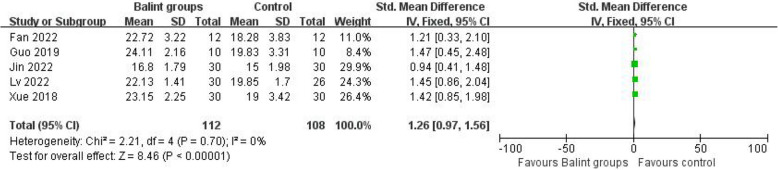
Effect of Balint-only groups on SEGUE score. The comparative result of *IV *Inverse variance, fixed effects meta-analysis between the Balint group and control group. *SD *Standard deviation, *CI *Confidence interval

The crude meta-analysis result showed that the SEGUE score was significantly higher in the Balint group compared with the control group (SMD = 2.31, 95% CI 1.29 to 3.33, *I*^2^ = 93%) (Fig. 2). Considering the co-interventions in two studies [17, 35], the analysis was conducted to separate these two studies from the others. The results showed that the SEGUE score in the Balint-only group was significantly higher than those in the placebo (SMD = 1.26, 95% CI 0.97 to 1.56, *I*^2^ = 0%) (Fig. 3).

#### Empathy score

**Fig. 4:**
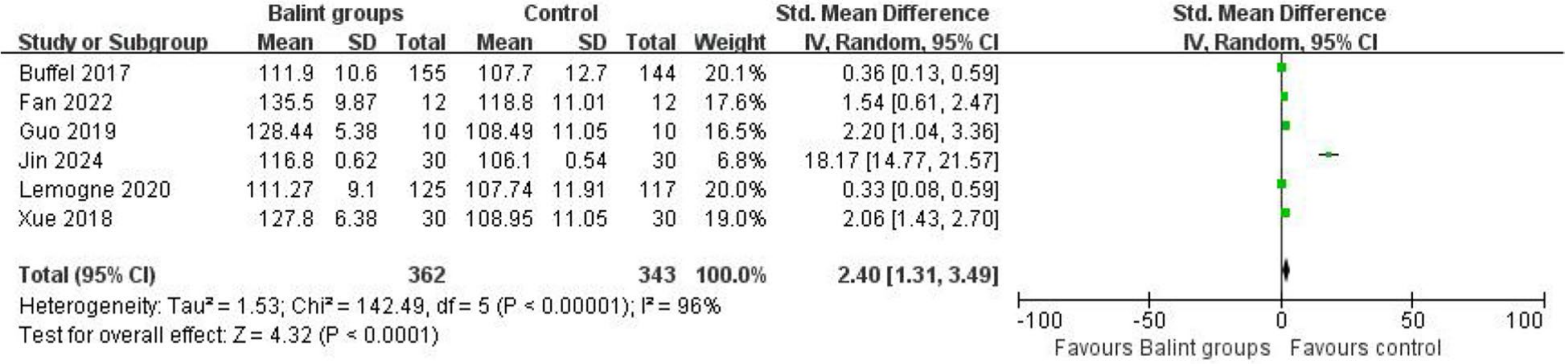
Effect of Balint groups on empathy score. The comparative result of *IV *Inverse variance, random effects meta-analysis between the Balint group and control group. *SD *Standard deviation, *CI *Confidence interval

Figure 3 shows that the Balint group had a favorable effect on the empathy score (SMD = 2.40, 95%, CI 1.31 to 3.49, *I*^2^ = 96%) (Fig. 4).

#### SAS score (anxiety score)

**Fig. 5:**
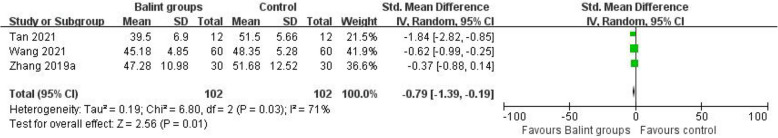
Effect of Balint groups on SAS score. The comparative result of *IV *Inverse variance, random effects meta-analysis between the Balint group and control group. *SD *Standard deviation, *CI *Confidence interval

Compared with the control group, the Balint group had a significant decrease in the SAS score (SMD = −0.79, 95% CI −1.39 to −0.19, *I*^2^ = 71%) (Fig. 5).

#### MBI-GS score (burnout score)

**Fig. 6:**
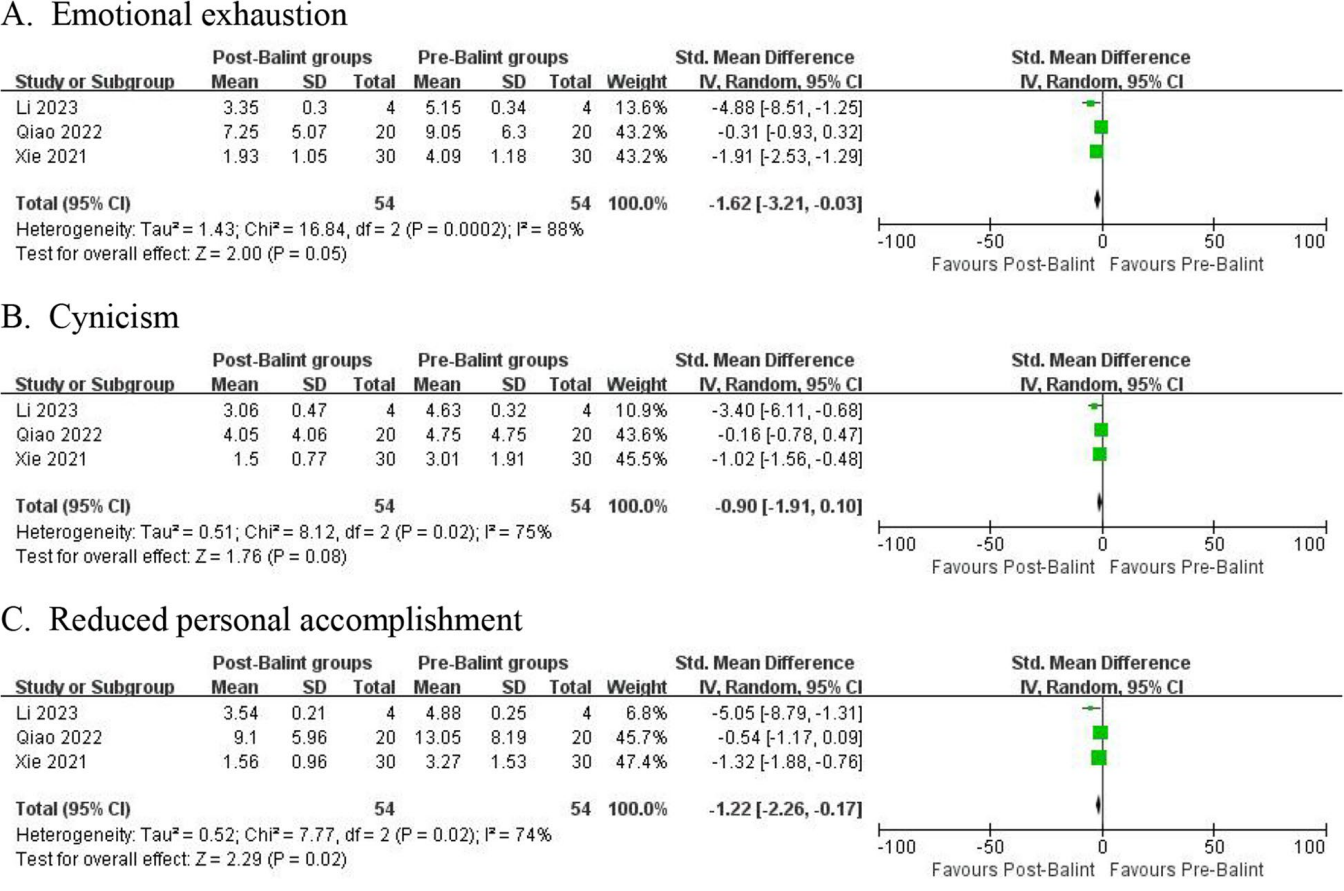
Effect of Balint groups on MBI-GS score. The comparative result of *IV *Inverse variance, random effects meta-analysis between the Balint group and control group. *SD* Standard deviation, *CI *Confidence interval

The meta-analysis results of single-arm trials showed the Balint group had a significant decrease in the MBI-GS score of emotional exhaustion and reduced personal accomplishment among healthcare workers (SMD = −1.62, 95% CI −3.21 to −0.03, *I*^2^ = 88%; SMD = −1.22, 95% CI −2.26 to −0.17, *I*^2^ = 74% respectively), while no significant change in cynicism (SMD = −0.90, 95%, CI −1.91 to 0.10, *I*^2^ = 75%) (Fig. 6).

#### Subgroup analysis and sensitivity analysis

The subgroup analysis results indicated that the type of participants and intervention measures significantly influenced the heterogeneity of the meta-analysis of SEGUE scores, with the resident group and co-interventions producing 95% and 99% heterogeneity, respectively. In contrast, neither the medical student group nor the Balint-only group showed significant heterogeneity. Meanwhile, study region and study quality significantly influenced the heterogeneity of the meta-analysis of empathy scores, and there was no significant heterogeneity in the studies conducted in France with lower risks compared to the 97% heterogeneity generated by the studies conducted in China with unclear bias. The meta-analysis of MBI-GS emotional exhaustion scores only involved three studies, with limited grouping variables, so the subgroup analysis failed to identify a clear source of heterogeneity (Supplementary Appendix 3).

The sensitivity analysis results showed change in the direction of the meta-analysis results of SEGUE scores and empathy scores. In contrast, the meta-analysis results of SAS and MBI-GS were not robust (Supplementary Appendix 4).

#### Publication bias

**Fig. 7:**
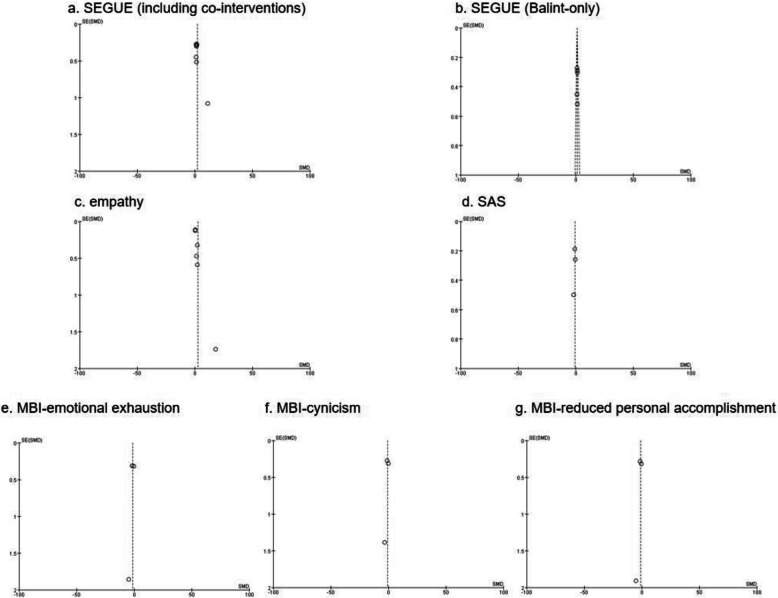
Publication bias of studies included in the meta-analysis

The publication bias of the meta-analyses included in this study is shown in Fig. 7. There was some publication bias in SEGUE score (including co-interventions) and empathy score.

### Meta-synthesis

**Fig. 8:**
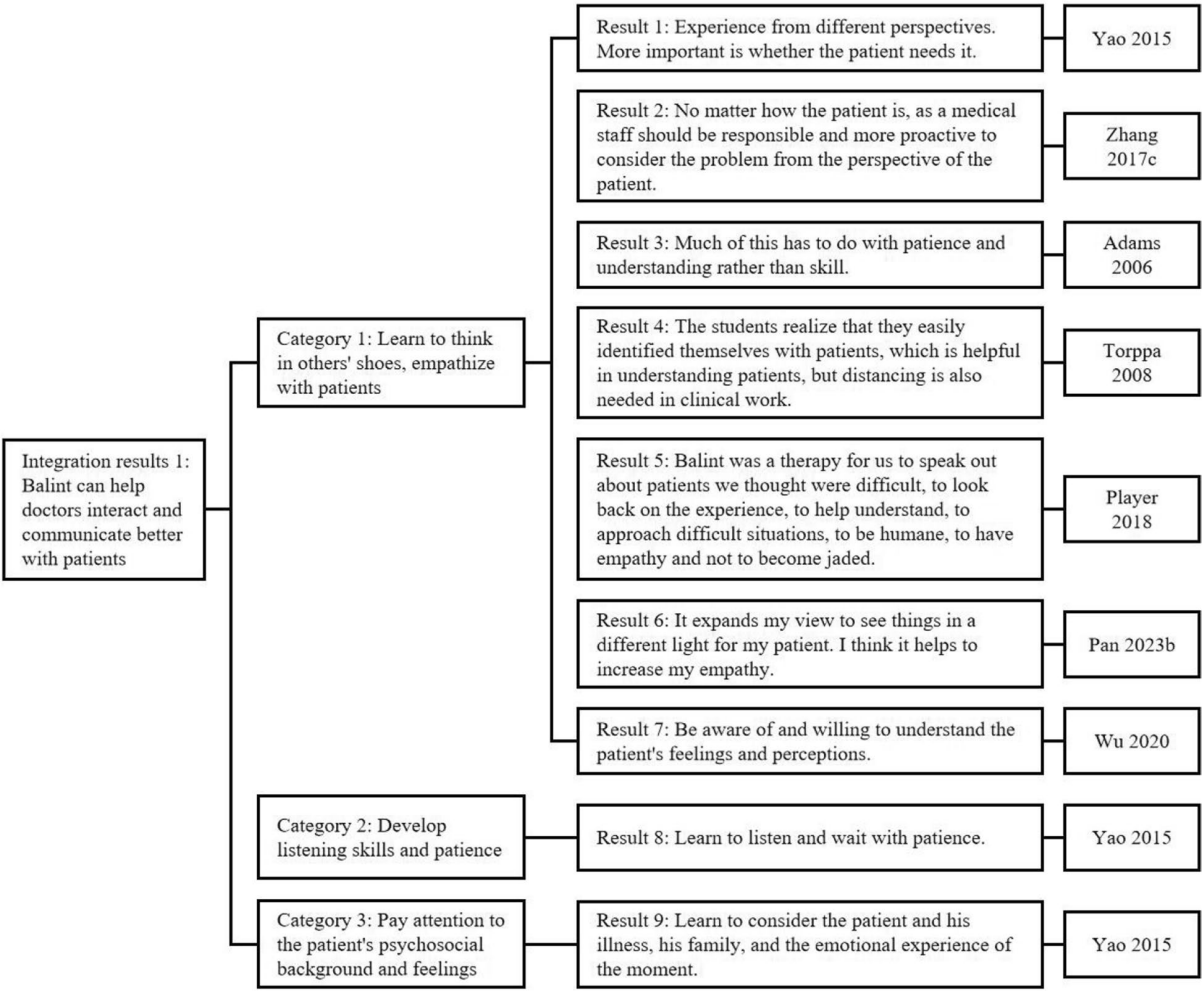
Integration process of the results of included studies (part 1)

**Fig. 9:**
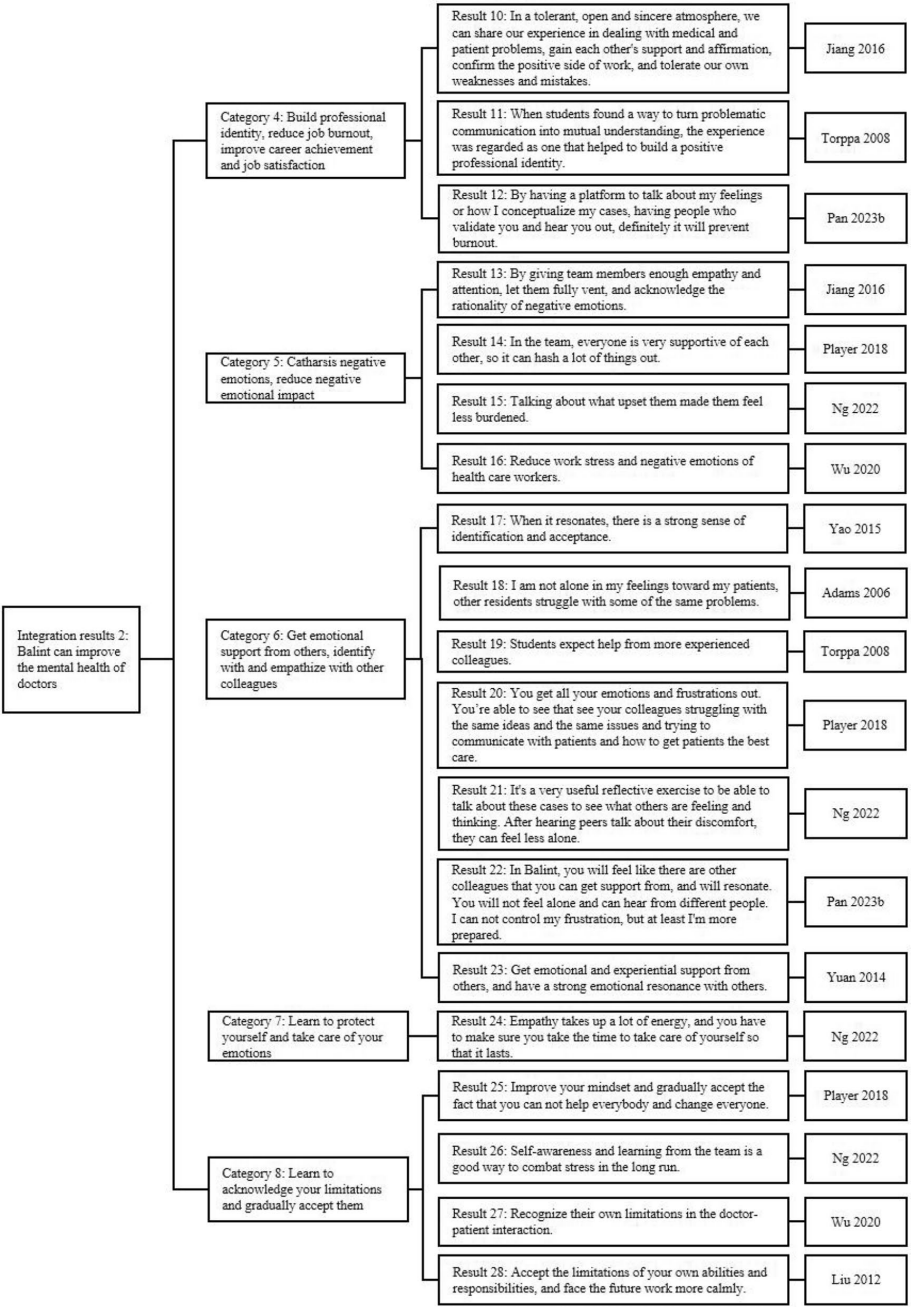
Integration process of the results of included studies (part 2)

**Fig. 10:**
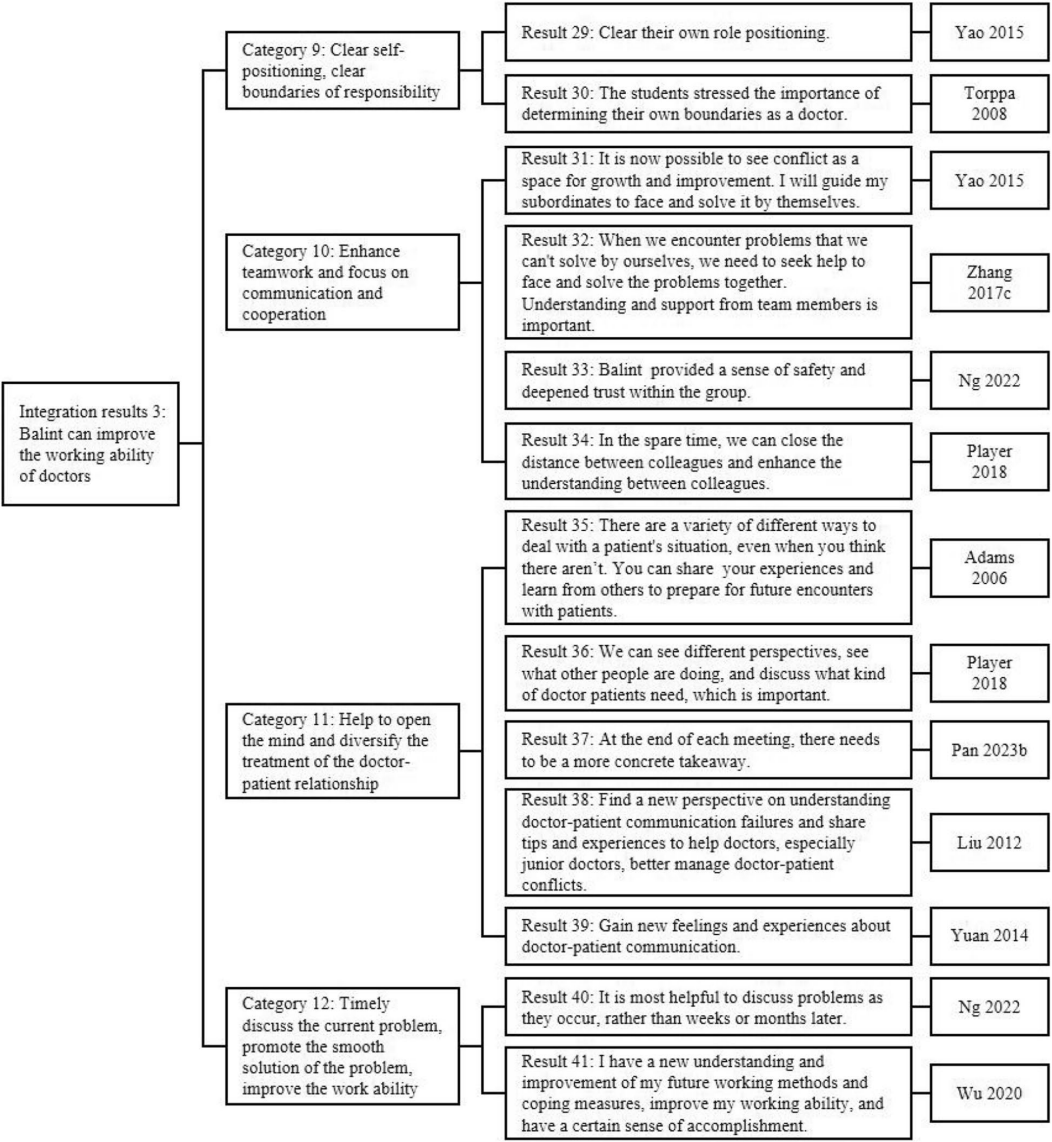
Integration process of the results of included studies (part 3)

A total of 11 qualitative studies were included in the meta-synthesis. After the researchers carefully read and analyzed these studies, 41 results were obtained, which were summarized into 12 categories; ultimately, 3 integrated results were obtained (Figs. 8, 9and 10). To summarize, Balint groups had a very positive effect on physicians’ ability to empathize with patients, mental health and regulation, teamwork, and problem-solving, with many of the participants having feelings and insights with respect to improving physicians’ mental health.

Most of the participants affirmed the role of the Balint group in helping doctors empathize with patients and believed that they could understand the position of patients more profoundly through discussions in the Balint group, combined with their own and other people’s experiences of practicing and providing medical care. This suggested that the Balint group played a role in helping doctors be patient with their patients, improving the doctor‒patient relationship, and reducing doctor‒patient conflicts.

With respect to mental health, most studies noted that Balint groups improved communication among colleagues, generating empathy and reflection while promoting the sharing of lessons learned with each other. This not only helped individuals to alleviate negative emotions but also helped them obtain emotional support and valuable work experience from others. This ultimately contributed to solving doctor‒patient problems at work, reducing burnout and increasing work satisfaction among physicians.

Additionally, the Balint group covered a wide range of topics during group discussions, allowing medical students, junior physicians, and senior physicians to discuss and share personal experiences from their daily work. This helped doctors develop the ability to think about and resolve conflicts between themselves and their patients, gain new insights, and attempt to solve problems.

## Discussion

To our knowledge, this is the first study to specifically focus on performing a comprehensive analysis of the intervention effects of Balint groups in doctor‒patient communication, including meta-analyses of quantitative studies and a meta-synthesis of qualitative studies. This study summarized the benefits of Balint groups, including enhancing the capacity of physicians or medical students to manage doctor‒patient relationships. A total of 56 intervention studies were included in this study, including 37 studies published in Chinese and 19 studies in English, involving a total of 3364 study participants. Among these, 13 studies were included in the quantitative meta-analysis, and 11 studies were included in the qualitative meta-synthesis. In conclusion, the meta-analysis and meta-synthesis results demonstrated that the Balint group has some certain effect on improving doctor‒patient relationships. Specifically, it can significantly enhance doctors’ communication skills, potentially improve empathy and team communication and cooperation, and may provide limited relief from alleviating occupational burnout and anxiety.

Overall, the designs of the Chinese and English studies differed greatly and had some commonalities. In terms of the intervention populations, since medical students and residents have little clinical experience and little communication with patients, the researchers focused more on medical students and residents than on physicians with clinical experience. This could be because medical students and residents need to learn from the experiences of others and improve their ability to address and cope with doctor‒patient relationships by participating in Balint groups [48, 67]; therefore, Balint groups can be used as an innovative method of medical education. In terms of the intervention, most studies only used Balint group interventions, while a few also used medical communication courses, music therapy [37], behavioral medicine training [23], and BBN training programs [29]. The studies were all controlled or had single-arm designs, with the number of participants ranging from a few to several hundred. The duration of the interventions varied from one month to one year.

In terms of outcome measurement, the Chinese researchers primarily used interviews and scales measuring empathy, burnout, anxiety, depression, and doctor‒patient communication ability to assess the role of Balint groups in improving doctor‒patient relationships. In contrast, researchers from other countries focused more on interviews, possibly because interviews can be more individualized and provide a deeper understanding of the effects of psychological interventions. Future research can focus on the ideal frequency and duration of interventions, evaluate which traits indicate the need for Balint group interventions, investigate what other psychological intervention therapies can be used in conjunction with Balint groups, and make greater efforts to standardize the measurement of outcome indicators and investigate the applicability of questionnaires.

Balint groups can help doctors better communicate with their patients, thus contributing to better doctor‒patient relationships. Building a positive patient‒physician relationship requires effective communication, particularly for interns or residents who are new to clinical practice and lack the experience and skills to communicate with patients. Balint groups can provide such learning opportunities for physicians or medical students, encouraging them to think critically and compensate for their flaws by learning from and understanding others [12]. In this study, the results of the meta-analysis of five controlled experiments and the meta-synthesis of results from seven qualitative studies revealed that Balint groups had a better effect on improving doctor–patient communication skills, which is in line with the findings of Yazdankhahfard et al. [68].

Empathy is one of the competencies that doctors must possess, as it affects how doctor–patient relationships are viewed and managed. Brock Brock et al. [69]; Player et al. [63] Doctors who are empathic can understand patients’ situations more deeply and provide them with more effective therapy. In the qualitative analysis, many included studies highlighted how the Balint group improved empathy by teaching participants to think differently and approach problems from different angles. The unique benefits of Balint groups in fostering empathy are primarily determined by the fact that, in contrast to general training approaches, Balint groups focus on participant’ personal experiences rather than typical cases derived from others, a feature that determines the special advantages of Balint groups in empathy development [13, 64]. Therefore, Balint groups can be used as a novel way to teach medical professionals or students how to manage doctor‒patient relationships. They accomplish this by having medical professionals or students discuss real cases of their peers from multiple perspectives, such as those of patients, doctors, and outsiders. This helps the participants better understand patients’ situations, which fosters the development of empathy [61]. However, it was possible that since the empathy scores were self-reported and there were too few studies included, the issue of statistical heterogeneity was prominent. In addition, the quality of the included studies varied, including several high-risk studies from China and two low-risk studies from France, resulting in significant shortcomings in the quality of the evidence. Therefore, this study conducted the meta-synthesis of qualitative studies, concluding that the Balint group can help doctors better understand patients’ feelings and perspectives, thereby partially supplementing the role of the Balint group in empathy.

The Balint group may be an effective way to alleviate occupational burnout and negative emotions among doctors, which can help them treat patients better and thereby ease the doctor-patient relationship. However, the reliability of the evidence in this study is limited. At present, medical personnel are facing heavy clinical workloads and increasingly tense doctor‒patient relationships while undertaking treatment, teaching, and research. A meta-analysis revealed that the detection rate of burnout among medical personnel in China was as high as 66% [70]. Several studies have demonstrated that long-term occupational stress causes damage to the mental health of medical personnel and a loss of professional enthusiasm, which can impact how well doctor‒patient relationships are managed and even the standard of medical care provided. This leads to a vicious circle of damage to physical and mental health as well as the relationships between doctors and patients [71]. The meta-analysis and meta-synthesis of this study revealed the potential role of Balint groups in alleviating burnout and reducing anxiety. However, due to the high heterogeneity of the included studies (including differences in intervention objectives, intervention methods, and study design quality) and the limited number of included studies, the quantitative evidence supporting the empathetic effects of Balint groups was insufficient. Therefore, we explored the results of qualitative studies and found that existing studies have pointed out the potential role of Balint groups in improving communication among colleagues and obtaining emotional support from colleagues. Existing articles also remain somewhat controversial. Several qualitative studies have demonstrated how Balint groups help enhance professional identity, achieve emotional resonance with colleagues, and regulate mental states. Nevertheless, some research has indicated that Balint groups do not significantly improve mental health or reduce stress [24]. This could be because of the various questionnaires used in the studies, and further research on the applicability of questionnaires could increase the caliber of the research. In general, Balint groups may help address mental health issues and reduce burnout in doctors; the evidence quality is insufficient to draw reliable conclusions, so higher-quality research is still needed in this area.

Balint groups can be used to explore the doctor‒patient communication problems encountered in clinical work. In addition to helping participants broaden their perspectives and find better solutions, these groups may also foster cooperation, provide opportunities for communication, and increase participants' abilities in many aspects. In this regard, the meta-synthesis in this study indicated that while qualitative research has reported some results, quantitative research has not yet explored teamwork and problem-solving abilities. Therefore, future research can focus on the role of Balint groups in this regard and examine the effect of Balint groups with a more scientific research design and more appropriate scales.

### Strengths and limitations

This is the first meta-analysis and meta-synthesis to examine the role of Balint groups in improving doctor–patient relationships. In conclusion, the present study mainly evaluated the effects from the perspectives of doctor–patient communication skills, empathy, and psychological well-being, which can provide a reference for future studies. In addition, compared with quantitative research, qualitative research is more helpful in revealing the mechanisms behind psychological therapies and can provide a more thorough and distinctive picture of their impacts. Thus, a meta-analysis and meta-synthesis were performed, and the results of the quantitative and qualitative research were combined in this study to more fully represent the impact of Balint groups.

However, this study also has certain limitations, which are reflected mainly in the following aspects. First, the scale of the intervention study was limited by the specificity of this method because Balint groups are small-group activities. The heterogeneity of meta-analyses was high due to small sample sizes and diverse types of study participants, which was confirmed by the results of the subgroup analyses, thus limiting the extrapolation of the results of this study. Second, most included studies, especially Chinese studies, had an ‘unclear’ risk of bias or led to ‘some concerns’ owing to the lack of detailed reporting on blinding and randomization. In particular, the risk of bias in most Chinese studies included in the meta-analyses was controversial. Therefore, for the empathy scores of both high-quality English studies and unclear-risk Chinese studies included simultaneously, we conducted subgroup analyses. Third, the length of the interventions in some of the included studies was unknown, making it difficult to conduct subgroup analyses of the effects of interventions with different durations. Fourth, the included studies used a wide variety of scales, some of which were self-administered, making it difficult to integrate quantitative data, and the vast majority of the studies relied on self-reports by physicians or medical students [68], which is, of course, related to the specificity of Balint groups. Fifth, the robustness of the results was impacted by substantial heterogeneity and publication bias in some meta-analyses, and the evidence quality was not high. Thus, this study performed subgroup analyses on this to reveal sources of heterogeneity, and the sensitivity analyses showed that the results were relatively robust. (Supplementary Appendix 4 and Supplementary Appendix 5) Sixth, this study only included Chinese and English studies. Due to language limitations, we have not searched and analyzed the studies in databases from other regions, which may have led to a certain degree of selection bias.

## Conclusions

This study illustrated how Balint groups significantly enhance doctors’ communication skills, potentially improve empathy and team communication and cooperation, and may provide limited relief from alleviating occupational burnout and anxiety from multiple perspectives of quantitative and qualitative studies. Future studies should conduct more multicenter large-sample RCTs with low risk-of-bias designs and stress the importance of qualitative studies in assessing the effects of interventions and designing more scientific and reasonable research protocols to increase the credibility of the study design and findings.

## Supplementary Information

Supplementary Material 1

Supplementary Material 2

Supplementary Material 3

Supplementary Material 4

Supplementary Material 5

Supplementary Material 6

## Data Availability

All data generated or analyzed in this study were obtained from published articles.

## References

[1] Mao Y, Xie T, Ning W. The influence of medical service quality on patients’ perception of doctor-patient relationship: mediating effect analysis based on patient satisfaction. J Xi’an Jiaotong Univ (Soc Sci). 2020;40:119–27. 10.15896/j.xjtuskxb.202006012.

[2] Lv X. From doctor-patient relationship governance to doctor-patient community construction: a coordinated approach to rebuilding doctor-patient trust. J Nanjing Normal Univ (Soc Sci Ed ). 2020:84–93.

[3] Sklar J. Regression and new beginnings: Michael, Alice and Enid Balint and the circulation of ideas. Int J Psychoanal. 2012;93:1017–34. 10.1111/j.1745-8315.2012.00559.x. 22900562 10.1111/j.1745-8315.2012.00559.x

[4] Yu Q, Chen H, Zheng Y. Application of Balint Group Practice in the standardized residency training. Chin J Grad Med Educ. 2019;3:423–5.

[5] Diaz VA, Chessman A, Johnson AH, Brock CD, Gavin JK. Balint groups in family medicine residency programs: a follow-up study from 1990–2010. Fam Med. 2015;47:367–72. 25905879

[6] Jiang W, Jia Y. The role of the Balint Group in promoting empathy in nurses. J Nurs Training. 2017;32:903–4. 10.16821/j.cnki.hsjx.2017.10.015.

[7] Lemogne C, Buffel Du Vaure C, Hoertel N, Catu-Pinault A, Limosin F, Ghasarossian C, et al. Balint groups and narrative medicine compared to a control condition in promoting students’ empathy. BMC Med Educ. 2020;20:412. 10.1186/s12909-020-02316-w. 33167952 10.1186/s12909-020-02316-w PMC7654605

[8] Page MJ, McKenzie JE, Bossuyt PM, Boutron I, Hoffmann TC, Mulrow CD, et al. The PRISMA 2020 statement: an updated guideline for reporting systematic reviews. BMJ. 2021;372:n71. 10.1136/bmj.n71. 33782057 10.1136/bmj.n71 PMC8005924

[9] Sterne J, Savovic J, Page MJ, Elbers RG, Blencowe NS, Boutron I, et al. RoB 2: a revised tool for assessing risk of bias in randomised trials. BMJ. 2019;366:l4898. 10.1136/bmj.l4898. 31462531 10.1136/bmj.l4898

[10] Wells GA, Shea B, O'Connell D, Peterson J, Welch V, Losos M PT. The Newcastle-Ottawa Scale (NOS) for assessing the quality of nonrandomised studies in meta-analyses. 2000. https://www.ohri.ca/programs/clinical_epidemiology/oxford.asp. Accessed 1 Jan 2024.

[11] Andrade C. Mean difference, Standardized Mean Difference (SMD), and their use in meta-analysis: as simple as it gets. J Clin Psychiatry. 2020;81. 10.4088/JCP.20f13681. 10.4088/JCP.20f13681 32965803

[12] Fan M, Gang J, Li C, Zhu W, Yang Z, Song C. A study on the improvement of empathy and doctor-patient communication ability of medical students during practice with Balint group activities. Health Vocational Educ. 2022;40:85–7. 10.20037/j.issn.1671-1246.2022.17.33.

[13] Xue C, Zhang H, Xu Z, Jie Y, Wang Z, Chen Y, et al. Effect of the Balint group on improving the empathy and doctor-patient communication skills among medical students inclinical practice. Chin J Med Educ Res. 2018;17:201–5. 10.3760/cma.j.issn.2095-1485.2018.02.023.

[14] Guo H, Zhang Q, Zhou Y, Zhu G. The influence of Balint group activities on empathic ability and communication ability of Chinese medicine residents in standardized training. J Trad Chin Med Manag. 2019;27:103–5. 10.16690/j.cnki.1007-9203.2019.12.052.

[15] Fu J, Fu J, Tao J, Zhang Y, Lin H, Xu K. A study on the application of empathy training in Balint group to improve the doctor-patient communication ability of otolaryngology head and neck surgery trainees. China High Med Educ. 2021:77-8. 10.3969/j.issn.1002-1701.2021.06.039.

[16] Jin X, Zheng M. The role of the Balint group in the practice of standardized training resident master. J Wenzhou Med Univ. 2022;52:337–40. 10.3969/j.issn.2095-9400.2022.04.017.

[17] Shao H, You C, Li Q, Wu P. Research on the application of Miller’s pyramid theory combined with Bahrain’s team activities in standardized residency training of burn surgeons. Chin J Med Educ Res. 2023;22:1230–3. 10.3760/cma.j.cn116021-20211201-01392.

[18] Xie F. The research on the effect of Balint groups for improving the empathy of medical students. AnHui Medical University; 2017.

[19] Wang T, Xie S, Xu Z, Shen J, Li W, Ji G. The improvement effect of Balint group activities on the anxiety of medical staff with medical disputes. J Mod Med Health. 2021;37:2985–7.

[20] Qiu Y, Li P, Song J, Ding L, You Y, Zhang Z. Evaluation of depression self-assessment by resident physicians in a tertiary general hospital and evaluation of the effect of intervention by Balint group. Chin Gen Pract. 2017;20:479–82.

[21] Zhang H, Fang Y, Ji M. Training effect of core skills course on doctor-patient communications in skills training to general practitioners. Mod Hosp. 2019;19:185–7. 10.3969/j.issn.1671-332X.2019.02.008.

[22] Tan, H. The mental health status and intervention research of medical workers in Qinghai Province. Qinghai Normal University; 2021.

[23] Turner AL, Malm RL. A preliminary investigation of balint and non-balint behavioral medicine training. Fam Med. 2004;36:114–22. 10.1016/S0095-4543(03)00123-4. 14872358

[24] Adams KE, O’Reilly M, Romm J, James K. Effect of balint training on resident professionalism. Am J Obstet Gynecol. 2006;195:1431–7. 10.1016/j.ajog.2006.07.042. 16996457 10.1016/j.ajog.2006.07.042

[25] Yang Z, Chen W. The randomized controlled trials of the Balint group to improve the physician-patient relationship in the intern group. China Contin Med Educ. 2017;9:14–5. 10.3969/j.issn.1674-9308.2017.27.007.

[26] Pan JY, Yong F, Chua TE, Chen HY. Tele-balint under the microscope: what really happens in tele-balint groups? Int J Psychiatry Med. 2023;58:231–48. 10.1177/00912174221092505. 35499173 10.1177/00912174221092505

[27] Yakeley J, Shoenberg P, Morris R, Sturgeon D, Majid S. Psychodynamic approaches to teaching medical students about the doctor–patient relationship: randomised controlled trial. Psychiatrist. 2011;35:308–13. 10.1192/pb.bp.110.033704.

[28] Zhang P, Luo J, Zhang H, Liu Y. Effect analysis on Balint group intervention in job burnout among community medical staff. Shanghai J Prev Med. 2019a;31:373–6. 10.19428/j.cnki.sjpm.2019.19048.

[29] Amiel GE, Ungar L, Alperin M, Baharier Z, Cohen R, Reis S. Ability of primary care physician’s to break bad news: a performance based assessment of an educational intervention. Patient Educ Couns. 2006;60:10–5. 10.1016/j.pec.2005.04.013. 16122897 10.1016/j.pec.2005.04.013

[30] Huang L, Harsh J, Cui H, Wu J, Thai J, Zhang X, et al. A randomized controlled trial of Balint groups to prevent burnout among residents in China. Front Psychiatry. 2020;10:957. 10.3389/fpsyt.2019.00957. 32116808 10.3389/fpsyt.2019.00957 PMC7026367

[31] Airagnes G, Consoli SM, De Morlhon O, Galliot A, Lemogne C, Jaury P. Appropriate training based on Balint groups can improve the empathic abilities of medical students: a preliminary study. J Psychosom Res. 2014;76:426–9. 10.1016/j.jpsychores.2014.03.005. 24745786 10.1016/j.jpsychores.2014.03.005

[32] Buffel DVC, Lemogne C, Bunge L, Catu-Pinault A, Hoertel N, Ghasarossian C, et al. Promoting empathy among medical students: A two-site randomized controlled study. J Psychosom Res. 2017;103:102–7. 10.1016/j.jpsychores.2017.10.008. 29167035 10.1016/j.jpsychores.2017.10.008

[33] Hang R, Cheng Z, Sheng X, Chen J, Lou J. Effect of balint-style group on empathy and interpersonal trust of medical students. Chin J Clin Psychol. 2017;25:783–8. 10.16128/j.cnki.1005-3611.2017.04.043.

[34] Gong Y, Shen J, Zhou Y. Application of Balint group model based on “unexpected diagnosis and treatment result” notification in doctor-patient communication teaching. Chin J Clin. 2024;52:877–9. 10.3969/j.issn.2095-8552.2024.07.034.

[35] Jin X, Lin X, Xiao J. The application of Balint group in doctor-patient communication course. J Wenzhou Med Univ. 2024;54:771–5. 10.3969/j.issn.2095-9400.2024.09.014.

[36] Lv L, Chen L, Peng Y, Fang H. Application of Balint group activities in improving the doctor-patient communication ability in standardized training of resident in department of burn. Mil Med Joint Logistics. 2022;36:729–32. 10.13730/j.issn.1009-2595.2022.09.012.

[37] Wan S, Wu Y, Zhang K. G.Balint’s application in improving doctor-patient communication skills of medical students. China Contin Med Educ. 2019;11:62–4. 10.3969/j.issn.1674-9308.2019.35.024.

[38] Sekeres MA, Chernoff M, Lynch TJ, Kasendorf EI, Lasser DH, Greenberg DB. The impact of a physician awareness group and the first year of training on hematology-oncology fellows. J Clin Oncol. 2003;21:3676–82. 10.1200/JCO.2003.12.014. 14512400 10.1200/JCO.2003.12.014

[39] Liu L. The effect of the Balint group training model in improving the doctor-patient communication skill in clinical medical students. Guangzhou Med J. 2019;50:113–6. 10.3969/j.issn.1000-8535.2019.05.028.

[40] Tan W, Xiao W, Lu X, Zhou W. Application of the “Balint group” in the doctor- patient communication skills training for standardized residency training of internal medicine. Chin J Med Educ Res. 2022;21:906–9. 10.3760/cma.j.cn116021-20200410-00931.

[41] Xie Y, Qiu X, Zhang A, Huang H, You L. Observe the effect of Balint group on relieving job burnout of psychiatrists. Psychologies Mag. 2021;16:13–4. 10.19738/j.cnki.psy.2021.09.006.

[42] Qiao S, Wu S, Yang S, Peng A, Zhu J. The influence of Balint group on job burnout and negative emotion of medical staff. Psychologies Mag. 2022;17:77–9. 10.19738/j.cnki.psy.2022.02.024.

[43] Li X, Wang Y, Chen P, Zhang S, Zhang W. The role of Balint group activities in improving empathic ability and communication ability of pediatric residents of traditional Chinese medicine. J Trad Chin Med Manag. 2023;31:93–5. 10.16690/j.cnki.1007-9203.2023.19.067.

[44] Qin C, Tan M, Liu Y. Effects of Balint group model on psychological crisis and coping styles of medical staffs in department of obstetrics and gynecology. China J Health Psychol. 2019;27:603–6. 10.13342/j.cnki.cjhp.2019.04.011.

[45] Pang J, Lu L, Chen C, Zhang X, Gu C, Bo Y. Influence of Balint group and relative training on the ability of physician-patient communication and coping style. Mil Nurs. 2015;32:60–3. 10.3969/j.issn.1008-9993.2015.20.016.

[46] Zou Y, Yang M, Yu J, Li Y, Cui Z. Practice and application of Balint group in improving doctor patient communication ability of interns. Guide Sci Educ. 2021:190–2. 10.16400/j.cnki.kjdk.2021.16.061.

[47] Zheng Y, Mo X, Yan Q, Tang X, Zhao Z, Xie Y. The effect of Balint’s group on the attitude of clinical interns to learning doctor-patient communication skills. Health Vocational Educ. 2020;38:108–10.

[48] Gajree N. Can Balint groups fill a gap in medical curricula? Clin Teach. 2021;18:158–62. 10.1111/tct.13298. 33073928 10.1111/tct.13298

[49] Zhang Y. The research and the education of clinical competence under the bio-psycho-social medical model. Jilin University; 2017a.

[50] Parker S, Leggett A. Teaching the clinical encounter in psychiatry: a trial of Balint groups for medical students. Australas Psychiatry. 2012;20:343–7. 10.1177/1039856212447965. 22767937 10.1177/1039856212447965

[51] Parker SD, Leggett A. Reflecting on our practice: an evaluation of Balint groups for medical students in psychiatry. Australas Psychiatry. 2014;22:190–4. 10.1177/1039856213517946. 24449530 10.1177/1039856213517946

[52] Dokter HJ, Duivenvoorden HJ, Verhage F. Changes in the attitude of general practitioners as a result of participation in a Balint group. Fam Pract. 1986;3:155–63. 10.1093/fampra/3.3.155. 3770335 10.1093/fampra/3.3.155

[53] Huang L, He W, Wang H, Cui H. Exploration and practice of Balint group in the standardized training of resident doctors. Shanghai Med Pharm J. 2017;38:3–6. 10.3969/j.issn.1006-1533.2017.22.002.

[54] Zhang P, Zhu X, Chen L, Zhang Y, Wu T, Deng B. Balint’s group combined the use of figure “sculpture” in the standardized training of residents. J Mod Med Health. 2017;33:291–3. 10.3969/j.issn.1009-5519.2017.02.050.

[55] Liu C, Xu Y, Luo Y, Zhou Y. Application of Balint group activities in the national standardized training of general practitioners. Chin Gen Pract. 2018;21:3858–62. 10.12114/j.issn.1007-9572.2018.31.017.

[56] Jiang L, Huang Z, Yang Y, Xu L. Clinical meaning of balint group in the skill training of medical personnel. Chin J Med. 2016;51:76–9. 10.3969/j.issn.1008-1070.2016.12.024.

[57] Wu Q. The clinical significance of Balint group in skill training of hospital medical staff. Electron J Pract Clin Nurs Sci. 2020;5:179–80.

[58] Pan X, Chen B, Yang W, Huang J. Analysis on application effect of Balint group in cultivation of physician-patient communication ability of medical interns. Chin Community Doctors. 2023;39:166–8. 10.3969/j.issn.1007-614x.2023.10.056.

[59] Zhang N, He J, Yi J, Liang Y. Improve pre-internship clinical communication skills in medical undergraduates with the Balint Group. Contin Med Educ. 2017;31:28–30. 10.3969/j.issn.1004-6763.2017.12.015.

[60] Torppa MA, Makkonen E, Mårtenson C, Pitkälä KH. A qualitative analysis of student Balint groups in medical education: contexts and triggers of case presentations and discussion themes. Patient Educ Couns. 2008;72:5–11. 10.1016/j.pec.2008.01.012. 18295432 10.1016/j.pec.2008.01.012

[61] Ng L, Seu C, Cullum S. Modelling vulnerability: qualitative study of the Balint process for medical students. BMC Med Educ. 2022;22:436. 10.1186/s12909-022-03508-2. 35668447 10.1186/s12909-022-03508-2 PMC9170339

[62] Yao L, Ye Z, Wu H, Cheng M. Practical application of Balint group in general hospital. Nurs Rehabil J. 2015;14:772–5. 10.3969/j.issn.1671-9875.2015.08.027.

[63] Player M, Freedy JR, Diaz V, Brock C, Chessman A, Thiedke C, et al. The role of Balint group training in the professional and personal development of family medicine residents. Int J Psychiatry Med. 2018;53:24–38. 10.1177/0091217417745289. 29235909 10.1177/0091217417745289

[64] Liu W, Ye C, Chen H, Ji J. Qualitative study of doctor Balint group cases in general hospital. Chin Ment Health J. 2012;26:91–5. 10.3969/j.issn.1000-6729.2012.02.006.

[65] Yang Z, Chen H, Liu W, Ye C, Liu S. Qualitative study of Balint group cases in teaching hospital. Chin J Behav Med Brain Sci. 2014;23:215–7. 10.3760/cma.j.issn.1674-6554.2014.03.007.

[66] Ghetti C, Chang J, Gosman G. Burnout, psychological skills, and empathy: balint training in obstetrics and gynecology residents. J Grad Med Educ. 2009;1:231–5. 10.4300/JGME-D-09-00049.1. 21975984 10.4300/JGME-D-09-00049.1 PMC2931236

[67] Van Roy K, Vanheule S, Inslegers R. Research on balint groups: a literature review. Patient Educ Couns. 2015;98:685–94. 10.1016/j.pec.2015.01.014. 25681874 10.1016/j.pec.2015.01.014

[68] Yazdankhahfard M, Haghani F, Omid A. The balint group and its application in medical education: a systematic review. J Educ Health Promot. 2019;8:124. 10.4103/jehp.jehp_423_18. 31334276 10.4103/jehp.jehp_423_18 PMC6615135

[69] Brock CD, Salinsky JV. Empathy: an essential skill for understanding the physician-patient relationship in clinical practice. Fam Med. 1993;25:245. 8319851

[70] Li X, Guo H, Xiao Y. Detection rate of burnout among medical staff in China: a meta-analysis. Mod Prev Med. 2021;48:1379–83.

[71] Li Y, Lu H, Liu Y. Research advance on job burnout of medical staff. Mod Prev Med. 2015;42:3489–92.

