# Supplementary material for: Balint groups for improving the ability of doctors and medical students to manage the doctor–patient relationship: a systematic review, quantitative meta-analysis and qualitative meta-synthesis of intervention studies
Source: BMC Med Educ. 2025 Nov 3;25:1534. doi: 10.1186/s12909-025-08072-z (PMC12581237; doi:10.1186/s12909-025-08072-z)
Supplement: Supplementary file 1 — Supplementary Material 1 [file 12909_2025_8072_MOESM1_ESM.docx]

**Supplementary Appendix 1. Search Strategy of the systemic review.**

| **No.** | **Database** | **Search date** | **Search strategy** | **Language** | **Results** |
| --- | --- | --- | --- | --- | --- |
| 1 | PubMed | 25/10/2024 | (("Balint group"[Title/Abstract]) OR ("Balint"[Title/Abstract]) OR ("Balint groups"[Title/Abstract])) AND ((Physician-Patient Relations[MeSH Terms]) OR ("doctor patient relationship"[Title/Abstract]) OR ("physician patient relationship"[Title/Abstract]) OR ("doctor patient interaction"[Title/Abstract]) OR ("physician patient interaction"[Title/Abstract]) OR ("doctor patient communication"[Title/Abstract]) OR ("physician patient communication"[Title/Abstract])) | English | 236 |
| 2 | EMBASE | 25/10/2024 | ('doctor patient relationship'/exp OR 'doctor patient contact' OR 'doctor patient relation' OR 'doctor patient relationship' OR 'hospital patient relationship' OR 'hospital-patient relations' OR 'patient doctor relation' OR 'patient doctor relationship' OR 'patient physician relation' OR 'patient physician relationship' OR 'patient staff relation' OR 'patient therapist relation' OR 'patient therapist relationship' OR 'physician patient relation' OR 'physician patient relationship' OR 'physician-patient relations' OR 'relation, doctor patient' OR 'therapist patient relation' OR 'therapist patient relationship') AND ('balint group'/exp OR 'balint group' OR 'balint therapy') | English | 198 |
| 3 | Cochrane | 25/10/2024 | #1 Balint group  #2 Balint groups  #3 Balint  #4 Balint therapy  #5 #1 OR #2 OR #3 OR #4  #6 Physician-Patient Relations [MeSH Terms,explored]  #7 #5 AND #6 | English | 3 |
| 4 | China National Knowledge Infrastructure (CNKI) | 25/10/2024 | SU%=('巴林特' + '巴林特小组' + '巴林特小组工作' + '巴林特小组训练模式' + '巴林特小组活动' + '巴林特小组模式') * ('医患关系' + '医患关系紧张' + '医患关系研究' + '医患关系改善' + '医患关系现状' + '医患关系问题' + '医患沟通' + '医患沟通能力' + '医患沟通培训' + '医患沟通技能' + '医患沟通教学' + '医患沟通教育')  *Translation: SU % =('Balint' + 'Balint group' + 'Balint group work' + 'Balint group training mode' + 'Balint group activity' + 'Balint group mode') * ('Doctor-patient relationship' + 'Doctor-patient relationship strained' + 'doctor-patient relationship research' + 'Doctor-patient relationship improvement' + 'Current situation of doctor-patient relationship' + 'Doctor-patient relationship problem' + 'Doctor-patient communication' + 'Doctor-patient communication ability' + 'Training of doctor-patient communication' + 'Doctor-patient communication skill' + 'Doctor-patient communication teaching" + "Doctor-patient communication education") | Chinese | 126 |
| 5 | WanFang | 25/10/2024 | 主题: (“巴林特”) and ((“医患关系”) or (“医患沟通”))  *Translation: Subject: (" Balint ") and ((" Doctor-patient relationship ") or (" Doctor-patient communication ") | Chinese | 112 |
| 6 | China Science and Technology Journal Database (CSTJ) | 25/10/2024 | (T=巴林特 or K=巴林特) and (T=医患关系 or K=医患关系 or T=医患沟通 or K=医患沟通)  *Translation: (T= Balint or K= Balint) and (T= doctor-patient relationship or K= doctor-patient relationship or T= doctor-patient communication or K= doctor-patient communication) | Chinese | 61 |
