# Supplementary material for: Balint groups for improving the ability of doctors and medical students to manage the doctor–patient relationship: a systematic review, quantitative meta-analysis and qualitative meta-synthesis of intervention studies
Source: BMC Med Educ. 2025 Nov 3;25:1534. doi: 10.1186/s12909-025-08072-z (PMC12581237; doi:10.1186/s12909-025-08072-z)
Supplement: Supplementary file 2 — Supplementary Material 2 [file 12909_2025_8072_MOESM2_ESM.docx]

**Supplementary Appendix 2. Quality assessment of the systemic review.**

**Table S1. Quality assessment of randomized controlled trials (RoB 2).**

| **Study ID** | **Bias arising from the randomization process** | **Bias due to deviations from intended interventions** | **Bias due to missing outcome data** | **Bias in measurement of the outcome** | **Bias in selection of the reported result** | **Overall bias** |
| --- | --- | --- | --- | --- | --- | --- |
| Yang 2017 | some concerns | some concerns | low risk | low risk | low risk | some concerns |
| Xie 2018 | some concerns | some concerns | low risk | low risk | low risk | some concerns |
| Zhang 2019a | some concerns | low risk | low risk | low risk | low risk | some concerns |
| Wang 2021 | some concerns | some concerns | low risk | low risk | low risk | some concerns |
| Tan 2021 | some concerns | some concerns | low risk | low risk | low risk | some concerns |
| Jin 2022 | some concerns | some concerns | low risk | some concerns | low risk | some concerns |
| Pan 2023a | some concerns | low risk | low risk | some concerns | low risk | some concerns |
| Fu 2021 | some concerns | low risk | low risk | some concerns | low risk | some concerns |
| Fan 2022 | some concerns | some concerns | low risk | some concerns | low risk | some concerns |
| Shao 2023 | some concerns | low risk | low risk | some concerns | low risk | some concerns |
| Guo 2019 | some concerns | low risk | low risk | some concerns | low risk | some concerns |
| Zhang 2019b | some concerns | low risk | low risk | low risk | low risk | some concerns |
| Qiu 2017 | some concerns | some concerns | low risk | low risk | low risk | some concerns |
| Xue 2018 | some concerns | low risk | low risk | some concerns | low risk | some concerns |
| Turner 2004 | some concerns | low risk | low risk | low risk | low risk | some concerns |
| Adams 2006 | some concerns | low risk | low risk | low risk | low risk | some concerns |
| Amiel 2006 | high risk | low risk | low risk | some concerns | low risk | high risk |
| Yakeley 2011 | some concerns | low risk | low risk | low risk | low risk | some concerns |
| Lemogne 2020 | low risk | low risk | low risk | low risk | low risk | low risk |
| Gong 2024 | some concerns | low risk | low risk | some concerns | low risk | some concerns |
| Jin 2024 | some concerns | low risk | low risk | some concerns | low risk | some concerns |
| Hang 2020 | some concerns | low risk | low risk | some concerns | low risk | some concerns |
| Airagnes 2014 | some concerns | low risk | low risk | some concerns | low risk | some concerns |
| Buffel 2017 | low risk | low risk | low risk | low risk | low risk | low risk |
| Hang 2017 | some concerns | some concerns | low risk | low risk | low risk | some concerns |

**Table S2. Quality assessment of cluster-randomized trials (RoB 2).**

| **Study ID** | **Bias arising from the randomization process** | **Risk of bias arising from the timing of identification or recruitment of participants** | **Bias due to deviations from intended interventions** | **Bias due to missing outcome data** | **Bias in measurement of the outcome** | **Bias in selection of the reported result** | **Overall bias** |
| --- | --- | --- | --- | --- | --- | --- | --- |
| Wan 2019 | some concerns | some concerns | some concerns | low risk | low risk | low risk | some concerns |
| Lv 2022 | high risk | some concerns | low risk | low risk | low risk | low risk | high risk |

**Table S3. Quality assessment of the crossover trial (RoB 2).**

| **Study ID** | **Bias arising from the randomization process** | **Risk of bias arising from period and carryover effects** | **Bias due to deviations from intended interventions** | **Bias due to missing outcome data** | **Bias in measurement of the outcome** | **Bias in selection of the reported result** | **Overall bias** |
| --- | --- | --- | --- | --- | --- | --- | --- |
| Sekeres 2003 | some concrens | some concrens | some concrens | low risk | low risk | low risk | some concrens |

**Table S4. Quality assessment of single-arm trials (the adapted NOS).**

| **Study ID** | **Representative of the cases** | **Ascertainment of exposure** | **Assessment of outcome** | **Was follow-up long enough for outcomes to occur?** | **Adequacy of follow up** | **Overall bias** |
| --- | --- | --- | --- | --- | --- | --- |
| Jiang 2016 | low | low | unclear | unclear | unclear | unclear |
| Huang 2017 | low | low | unclear | low | low | unclear |
| Zhang 2017a | low | low | unclear | unclear | low | unclear |
| Zhang 2017b | low | low | unclear | unclear | unclear | unclear |
| Zhang 2017c | low | low | unclear | low | unclear | unclear |
| Liu 2018 | low | low | unclear | low | low | unclear |
| Liu 2019 | low | low | unclear | low | low | unclear |
| Qin 2019 | low | low | unclear | low | unclear | unclear |
| Zheng 2020 | low | low | unclear | unclear | low | unclear |
| Wu 2020 | low | low | unclear | unclear | unclear | unclear |
| Xie 2021 | low | low | unclear | low | low | unclear |
| Zou 2021 | low | low | unclear | unclear | low | unclear |
| Tan 2022 | low | low | unclear | low | low | unclear |
| Qiao 2022 | low | low | unclear | low | low | unclear |
| Li 2023 | low | low | unclear | unclear | low | unclear |
| Pang 2015 | low | low | unclear | high | unclear | high |
| Yao 2015 | low | low | unclear | low | unclear | unclear |
| Liu 2012 | low | low | unclear | low | unclear | unclear |
| Yang 2014 | low | low | unclear | unclear | unclear | unclear |
| Dokter 1986 | low | low | unclear | unclear | low | unclear |
| Torppa 2008 | low | low | unclear | unclear | low | unclear |
| Parker 2012 | low | low | unclear | low | unclear | unclear |
| Parker 2014 | low | low | unclear | unclear | unclear | unclear |
| Player 2018 | low | low | unclear | low | low | unclear |
| Gajree 2021 | low | low | unclear | low | low | unclear |
| Ng 2022 | low | low | unclear | low | low | unclear |
| Pan 2023b | low | low | unclear | unclear | low | unclear |
| Ghetti 2009 | low | low | unclear | high | high | high |
