# Supplementary material for: Balint groups for improving the ability of doctors and medical students to manage the doctor–patient relationship: a systematic review, quantitative meta-analysis and qualitative meta-synthesis of intervention studies
Source: BMC Med Educ. 2025 Nov 3;25:1534. doi: 10.1186/s12909-025-08072-z (PMC12581237; doi:10.1186/s12909-025-08072-z)
Supplement: Supplementary file 3 — Supplementary Material 3 [file 12909_2025_8072_MOESM3_ESM.docx]

**Supplementary Appendix 3. Subgroup analysis of the effect** **of Balint groups on SEGUE score, empathy score, and MBI-GS emotional exhaustion score.**

1. SEGUE score: Subgroup analyses were performed based on participants (Fig. S1), intervention measures (Fig. S2), and risk of bias (Fig. S3).


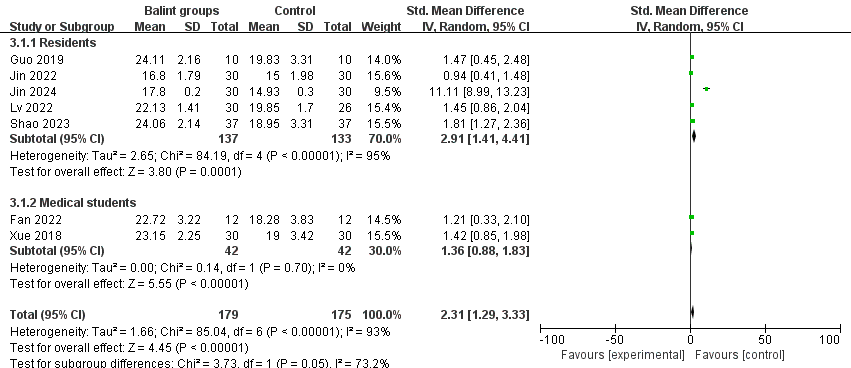


Fig. S1. Subgroup analysis of Balint groups on SEGUE score according to participants. Inverse variance (IV); SD, standard deviation; CI, confidence interval.


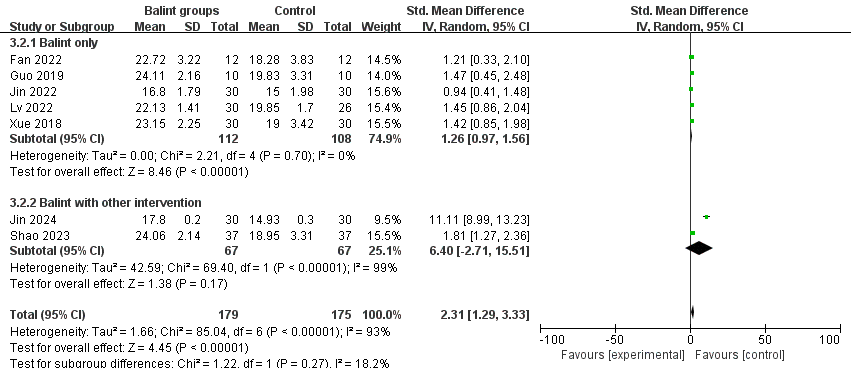


Fig. S2. Subgroup analysis of Balint groups on SEGUE score according to intervention measures of intervention groups. Inverse variance (IV); SD, standard deviation; CI, confidence interval.


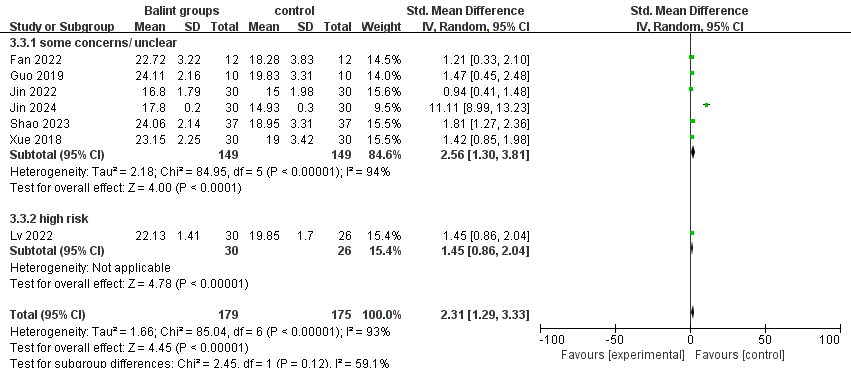


Fig. S3. Subgroup analysis of Balint groups on SEGUE score according to risk of bias. Inverse variance (IV); SD, standard deviation; CI, confidence interval.

1. Empathy score: Subgroup analyses were performed based on regions/languages (Fig. S4), risk of bias (Fig. S5), participants (Fig. S6), intervention (Fig. S7).


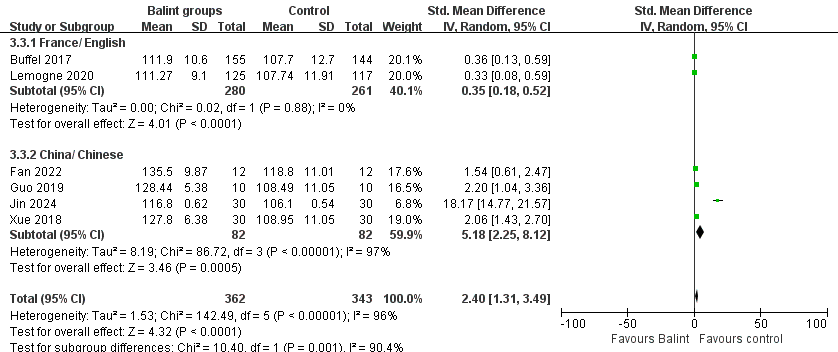


Fig. S4. Subgroup analysis of Balint groups on empathy score according to regions/ languages. Inverse variance (IV); SD, standard deviation; CI, confidence interval.


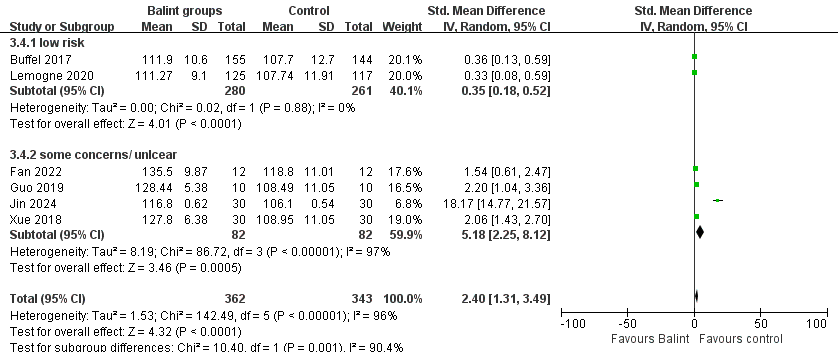


Fig. S5. Subgroup analysis of Balint groups on empathy score according to risk of bias. Inverse variance (IV); SD, standard deviation; CI, confidence interval.


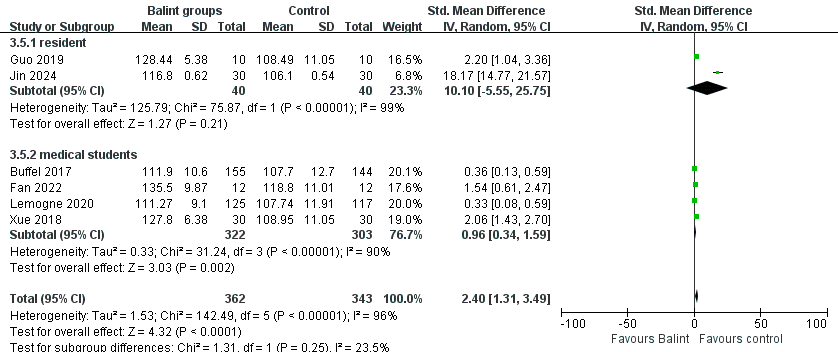


Fig. S6. Subgroup analysis of Balint groups on empathy score according to participants. Inverse variance (IV); SD, standard deviation; CI, confidence interval.


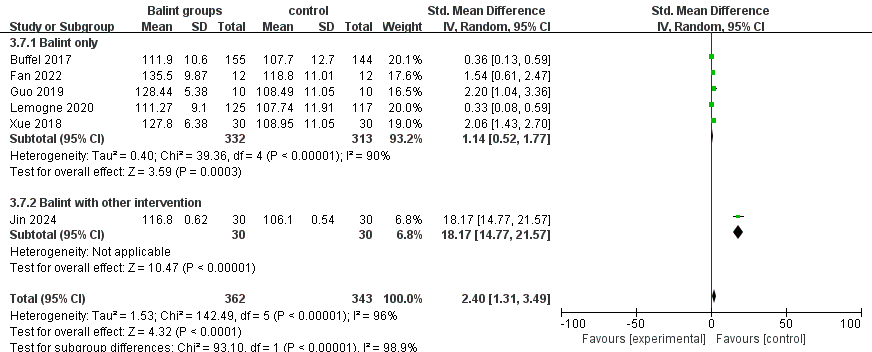


Fig. S7. Subgroup analysis of Balint groups on empathy score according to intervention. Inverse variance (IV); SD, standard deviation; CI, confidence interval.

1. MBI-GS emotional exhaustion score: Subgroup analysis was performed based on participants (Fig. S8).


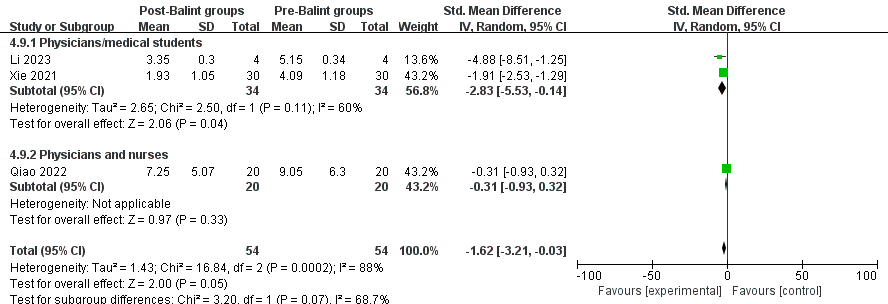


Fig. S8. Subgroup analysis of Balint groups on MBI-GS emotional exhaustion score according to participants. Inverse variance (IV); SD, standard deviation; CI, confidence interval.
