# Supplementary material for: Balint groups for improving the ability of doctors and medical students to manage the doctor–patient relationship: a systematic review, quantitative meta-analysis and qualitative meta-synthesis of intervention studies
Source: BMC Med Educ. 2025 Nov 3;25:1534. doi: 10.1186/s12909-025-08072-z (PMC12581237; doi:10.1186/s12909-025-08072-z)
Supplement: Supplementary file 4 — Supplementary Material 4 [file 12909_2025_8072_MOESM4_ESM.docx]

**Supplementary Appendix 4. Sensitivity analysis.**

Table S9. Sensitivity analysis for meta-analysis of SEGUE scores.

| Study omitted | SMD | lower CI | higher CI |
| --- | --- | --- | --- |
| Fan 2022 | 1.27 | 0.96 | 1.58 |
| Guo 2019 | 1.24 | 0.94 | 1.55 |
| Jin 2022 | 1.40 | 1.05 | 1.75 |
| Lv 2022 | 1.20 | 0.87 | 1.54 |
| Xue 2018 | 1.21 | 0.87 | 1.55 |

The Balint groups in (Jin et al., 2024; Shao et al., 2023) were combined with other effective interventions, so these two studies were excluded from the sensitivity analysis.

Table S10. Sensitivity analysis for meta-analysis of empathy scores.

| Study omitted | SMD | lower CI | higher CI |
| --- | --- | --- | --- |
| Buffel 2017 | 3.77 | 1.79 | 5.76 |
| Fan 2022 | 2.63 | 1.40 | 3.85 |
| Guo 2019 | 2.44 | 1.25 | 3.63 |
| Jin 2024 | 1.14 | 0.52 | 1.77 |
| Lemogne 2020 | 3.76 | 1.80 | 5.72 |
| Xue 2018 | 2.47 | 1.27 | 3.67 |

Table S11. Sensitivity analysis for meta-analysis of SAS score.

| Study omitted | SMD | lower CI | higher CI |
| --- | --- | --- | --- |
| Tan 2021 | -0.54 | -0.83 | -0.24 |
| Wang 2021 | -1.04 | -2.47 | 0.39 |
| Zhang 2019a | -1.14 | -2.32 | 0.04 |

Table S12. Sensitivity analysis for meta-analysis of MBI-GS score.

| Study omitted | SMD (95% CI) | | |
| --- | --- | --- | --- |
|  | emotional exhaustion | cynicism | reduced personal accomplishment |
| Li 2023 | -1.11 (-2.68, 0.46) | -0.60 (-1.45, 0.25) | -0.94 (-1.71, -0.18) |
| Qiao 2022 | -2.83 (-5.53, -0.14) | -1.82 (-4.02, 0.38) | -2.71 (-6.24, 0.82) |
| Xie 2021 | -1.91 (-2.53, -1.29) | -1.49 (4.62, 1.63) | -2.40 (-6.75, 1.95) |

References:

Jin, X., Lin, X., Xiao, J., 2024. The application of Balint group in doctor-patient communication course. Journal of Wenzhou Medical University. 54, 771-5. https://doi.org/10.3969/j.issn.2095-9400.2024.09.014.

Shao, H., You, C., Li, Q., Wu, P., 2023. Research on the application of Miller's pyramid theory combined with Bahrain's team activities in standardized residency training of burn surgeons. Chinese Journal of Medical Education Research. 22, 1230-3. https://doi.org/10.3760/cma.j.cn116021⁃20211201⁃01392.
