# Supplementary material for: Balint groups for improving the ability of doctors and medical students to manage the doctor–patient relationship: a systematic review, quantitative meta-analysis and qualitative meta-synthesis of intervention studies
Source: BMC Med Educ. 2025 Nov 3;25:1534. doi: 10.1186/s12909-025-08072-z (PMC12581237; doi:10.1186/s12909-025-08072-z)
Supplement: Supplementary file 5 — Supplementary Material 5 [file 12909_2025_8072_MOESM5_ESM.docx]

| Table S13. Grade assessment of meta-analyses (Balint groups vs control).  **Bibliography: Balint groups for improving doctor-patient communication skills in doctors/ medical students.** | | | | | | | | | | | |
| --- | --- | --- | --- | --- | --- | --- | --- | --- | --- | --- | --- |
| **Certainty assessment** | | | | | | | **Summary of findings** | | | | |
| **Participants (studies) Follow-up** | **Risk of bias** | **Inconsistency** | **Indirectness** | **Imprecision** | **Publication bias** | **Overall certainty of evidence** | **Study event rates (%)** | | **Relative effect (95% CI)** | **Anticipated absolute effects** | |
|  |  |  |  |  |  |  | **With control** | **With Balint groups** |  | **Risk with control** | **Risk difference with Balint groups** |
| **SEGUE score (co-interventions)** | | | | | | | | | | | |
| 354 (7 RCTs) | serious^a^ | not serious | not serious | not serious | publication bias strongly suspected^b^ | ⨁⨁◯◯ Low^a,b^ | 175 | 179 | - | - | SMD **2.31 SD higher** (1.29 higher to 3.33 higher) |
| **SEGUE score (Balint-only)** | | | | | | | | | | | |
| 220 (5 RCTs) | serious^a^ | not serious | not serious | not serious | none | ⨁⨁⨁◯ Moderate^a^ | 108 | 112 | - | - | SMD **1.26 SD higher** (0.97 higher to 1.56 higher) |
| **Empathy score** | | | | | | | | | | | |
| 705 (6 RCTs) | not serious | not serious | not serious | serious^c^ | publication bias strongly suspected^b^ | ⨁⨁◯◯ Low^b,c^ | 343 | 362 | - | - | SMD **2.4 SD higher** (1.31 higher to 3.49 higher) |
| **SAS score** | | | | | | | | | | | |
| 204 (3 RCTs) | not serious | not serious | not serious | serious^c^ | none | ⨁⨁⨁◯ Moderate^c^ | 102 | 102 | - | - | SMD **0.79 SD lower** (1.39 lower to 0.19 lower) |

**CI:** confidence interval; **SMD:** standardised mean difference

#### Explanations

a. The risk of bias of (Lv 2022) was high.

b. The funnel plot showed publication bias in the results.

c. The results were obtained by subjective evaluation.

Table S14. Grade assessment of meta-analyses (Post-Balint groups vs Pre-Balint groups).

| **Post-Balint groups vs Pre-Balint groups**  **Bibliography: . Balint groups for improving doctor-patient communication skills in doctors/ medical students.** | | | | | | | | | | | |
| --- | --- | --- | --- | --- | --- | --- | --- | --- | --- | --- | --- |
| **Certainty assessment** | | | | | | | **Summary of findings** | | | | |
| **Participants (studies) Follow-up** | **Risk of bias** | **Inconsistency** | **Indirectness** | **Imprecision** | **Publication bias** | **Overall certainty of evidence** | **Study event rates (%)** | | **Relative effect (95% CI)** | **Anticipated absolute effects** | |
|  |  |  |  |  |  |  | **With Pre-Balint groups** | **With Post-Balint groups** |  | **Risk with Pre-Balint groups** | **Risk difference with Post-Balint groups** |
| **MBI-GS score-emotional exhaustion** | | | | | | | | | | | |
| 108 (3 RCTs) | not serious | not serious | not serious | serious^a^ | none | ⨁⨁⨁◯ Moderate^a^ | 54 | 54 | - | - | SMD **1.62 SD lower** (3.21 lower to 0.03 lower) |
| **MBI-GS score-cynicism** | | | | | | | | | | | |
| 108 (3 RCTs) | not serious | not serious | not serious | serious^a^ | none | ⨁⨁⨁◯ Moderate^a^ | 54 | 54 | - | - | SMD **0.9 SD lower** (1.91 lower to 0.1 higher) |
| **MBI-GS score-reduced personal accomplishment** | | | | | | | | | | | |
| 108 (3 RCTs) | not serious | not serious | not serious | serious^a^ | none | ⨁⨁⨁◯ Moderate^a^ | 54 | 54 | - | - | SMD **1.22 SD lower** (2.26 lower to 0.17 lower) |

**CI:** confidence interval; **SMD:** standardised mean difference

#### Explanations

a. The results were obtained by subjective self-evaluation.
